# Economic evaluation of community-based falls prevention interventions for older populations: a systematic methodological overview of systematic reviews

**DOI:** 10.1186/s12913-022-07764-2

**Published:** 2022-03-26

**Authors:** Joseph Kwon, Hazel Squires, Matthew Franklin, Yujin Lee, Tracey Young

**Affiliations:** 1 School of Health and Related Research, University of Sheffield, Regent Court (ScHARR), 30 Regent Street, Sheffield, England S1 4DA; 2 Warwick Medical School, University of Warwick, Gibbet Hill Road, Coventry, England CV4 7AL

**Keywords:** Falls prevention, Economic evaluation, Systematic overview

## Abstract

**Background:**

Falls impose significant health and economic burdens on older people. The volume of falls prevention economic evaluations has increased, the findings from which have been synthesised by systematic reviews (SRs). Such SRs can inform commissioning and design of future evaluations; however, their findings can be misleading and incomplete, dependent on their pre-specified criteria. This study aims to conduct a systematic overview (SO) to: (1) systematically identify SRs of community-based falls prevention economic evaluations; (2) describe the methodology and findings of SRs; (3) critically appraise the methodology of SRs; and (4) suggest commissioning recommendations based on SO findings.

**Methods:**

The SO followed the PRISMA guideline and the Cochrane guideline on SO, covering 12 databases and grey literature for the period 2003–2020. Eligible studies were SRs with 50% or more included studies that were economic evaluations of community-based falls prevention (against any comparator) for older persons (aged 60 +) or high-risk individuals aged 50–59. Identified SRs’ aims, search strategies and results, extracted data fields, quality assessment methods/results, and commissioning and research recommendations were synthesised. The comprehensiveness of previous SRs’ data synthesis was judged against criteria drawn from literature on falls prevention/public health economic evaluation. Outcomes of general population, lifetime decision models were re-analysed to inform commissioning recommendations. The SO protocol is registered in the Prospective Register of Systematic Reviews (CRD42021234379).

**Results:**

Seven SRs were identified, which extracted 8 to 33 data fields from 44 economic evaluations. Four economic evaluation methodological/reporting quality checklists were used; three SRs narratively synthesised methodological features to varying extent and focus. SRs generally did not appraise decision modelling features, including methods for characterising dynamic complexity of falls risk and intervention need. Their commissioning recommendations were based mainly on cost-per-unit ratios (e.g., incremental cost-effectiveness ratios) and neglected aggregate impact. There is model-based evidence of multifactorial and environmental interventions, home assessment and modification and Tai Chi being cost-effective but also the risk that they exacerbate social inequities of health.

**Conclusions:**

Current SRs of falls prevention economic evaluations do not holistically inform commissioning and evaluation. Accounting for broader decisional factors and methodological nuances of economic evaluations, particularly decision models, is needed.

**Supplementary Information:**

The online version contains supplementary material available at 10.1186/s12913-022-07764-2.

## Background

An ageing population with increased prevalence of falls in older age (e.g., aged 60 + years) has made falls prevention a global public health priority [1]. Falls can cause mortality and substantial morbidity burden on older people including fear of falling [2], depression [3], functional decline [4], and fatality from serious injuries [5] with high care system costs [6, 7] and wider societal burden (i.e., informal caregiver burden and declined social interaction) [8, 9].

Falls prevention interventions have been found to be effective in reducing the number of falls and fallers in community settings [10–12]. Accordingly, cost-effectiveness evidence from falls prevention economic evaluations has grown; the most recent Cochrane review of randomized controlled trials (RCTs) identified 12 economic evaluations for community-based falls prevention exercise alone [12].

The rising volume of economic evaluations has been accompanied by systematic reviews of available evidence. For a well-formulated research question, a systematic review uses systematic and explicit methods to identify relevant studies, synthesise relevant extracted data, and critically appraise their quality [13, 14].

Two central functions of systematic reviews of economic evaluations can be: (A) to inform commissioning decisions; and (B) to summarise and evaluate the methodological features of economic evaluation in a topic area. Related to (A), the reviews can aid commissioning decisions by summarising the evaluation results most applicable to the decision-making context and/or identifying existing decision models that can be adapted and re-used [15]. In England and Wales, the development of the National Institute for Health and Care Excellence’s (NICE’s) falls prevention guideline (version CG21, later updated as CG161 [16]) involved a systematic review of falls prevention economic evaluations [17].

Related to (B), systematic reviews can detail and critically appraise methodological features that significantly affect the evaluation results such as the identification, measurement and valuation of all relevant costs and consequences and structural assumptions made by decision models [15, 18]. The appraisal could apply a pre-established checklist for methodological/reporting quality such as the Consolidated Health Economic Evaluation Reporting Standards (CHEERS) [19] and/or narratively synthesise methodological strengths and limitations. The findings from the methodological appraisal would also facilitate the conceptualisation of future economic evaluations, particularly decision models, and the identification of relevant data sources [15]. Additionally, and related to both (A) and (B), such appraisal would enable commissioners to consider the wide range of methodological factors that may qualify the evaluation results before applying them to the decision problem.

A systematic overview uses explicit and systematic methods to identify previous systematic reviews in a topic area [20]. It thus provides the highest level of economic evidence that can inform commissioning decisions as well as the opportunity for critically appraising the methodology of previous systematic reviews, specifically regarding how well they have performed the above functions (A) and (B). This would improve the methodological quality of: (i) future systematic reviews in the topic area; (ii) commissioning decisions based on the reviews; and (iii) future economic evaluations that utilise the reviews to conceptualise and implement their methodologies. The systematic overview is hence of interest both to consumers of economic evidence (i.e., commissioners, falls prevention professionals and patient groups) and to methodologists (i.e., systematic reviewers and falls prevention evaluators and modellers).

### Aim and objectives

The aim is to conduct a systematic overview of previous systematic reviews of community-based falls prevention interventions. The objectives are to:

1. Systematically search for and identify previous systematic reviews of community-based falls prevention economic evaluations;
2. Describe the methods and findings of previous systematic reviews, including their aim, search strategy and results, data extracted, quality assessment and commissioning and research recommendations;
3. Critically appraise the methodology of previous systematic reviews and highlight areas of improvement for future systematic reviews;
4. Suggest commissioning recommendations for falls prevention interventions based on syntheses of results and methodological quality of economic evaluations identified by systematic overview.

## Methods

The systematic overview followed the Cochrane guideline on overview of reviews [20] and the Preferred Reporting Items for Systematic Reviews and Meta-Analyse (PRISMA) 2020 guideline [13]. See Supplementary material for the PRISMA checklist. The review protocol is registered in the Prospective Register of Systematic Reviews (CRD42021234379).

### Search strategy and selection criteria

The search covered the period between January 2003 and December 2020 and 12 academic databases: Medline, Embase, PubMed, CDSR, CENTRAL, EconLit, CINAHL, PsycInfo, ASSIA, CRD, CEA Registry and PEDro. Grey literature studies were searched from online sites of Department of Health, Chartered Society of Physiotherapy, College of Occupational Therapy, Royal College of Nursing and Age UK. The start date was chosen based on a background knowledge that the number of economic evaluations before 2003 is low [17]. The search strategy was an intersection between terms for falls, terms for older people and terms for economic evaluation. All database search strategies are given in Tables A1-A8 and related texts in Supplementary material. References and citations of included studies were also searched.

Two researchers independently reviewed the titles and abstracts of identified articles at the first stage and the full texts of approved article at the second stage. Those that received two second-stage approvals were included for data extraction. Another researcher arbitrated in case of disagreement.

Included studies must have conducted a systematic review – i.e., involving the use of explicit, reproducible methodology, comprehensive search strategy and acceptable methods for data extraction and validity assessment of included studies by two or more researchers [20]. Additionally, more than 50% of the review’s included studies must have all of the following characteristics: (i) target population of community-dwelling (i.e., not in institutional settings that provide residential health and/or social care, such as inpatient wards and nursing homes) older persons (aged 60 +) and/or individuals aged 50–59 who are at high falls risk, from any country or sub- or trans-national regions; (ii) any intervention designed to reduce the number of falls or fall-related injuries, excluding specific disease rehabilitation (e.g., for stroke) with minor falls prevention component; (iii) any comparator(s); (iv) conduct full economic evaluations (i.e., comparative analyses of interventions in terms of their relative costs and consequences [18]), including single-vehicle evaluations (SVEs) (e.g., alongside RCTs) and decision models; and (v) full text in English.

### Data extraction and synthesis

Following the Cochrane guideline [20], the following data were extracted from the included reviews by two reviewers and narratively synthesised: (1) author(s), publication year and review aim; (2) search strategy and results – period, databases, eligible study designs, eligible interventions, other eligibility criteria, and number of economic evaluations identified; (3) reference and characteristics of economic evaluations identified by reviews; (4) data fields extracted from economic evaluations by reviews; (5) methods for quality assessment of economic evaluations by reviews and assessment results; and (6) commissioning and research recommendations made by reviews.

### Critical appraisal of previous systematic review methodology

As recommended by the Cochrane guideline [20], the 16-item AMSTAR 2 checklist [21] was applied independently by two reviewers to assess the reporting and methodological qualities of previous systematic reviews. Items 2, 9 and 13 in the AMSTAR 2 checklist that concerned the systematic reviews’ risk of bias assessment of included evaluations were expanded to concern the reviews’ broader methodological quality assessment of the evaluations, i.e., category (5) of extracted data above. This was because risk of bias in effectiveness estimation is only one of many factors determining the evaluation credibility, albeit an important one. For item 8 that concerned whether the reviews extracted ‘adequate detail’ from the economic evaluations, the number of data fields in Table 1 (described below) extracted by the reviews was used to score the item.

**Table 1:** Key data fields that should be extracted and narratively synthesised by systematic reviews of falls prevention economic evaluations

Category	Data field
(A) Setting, population and evaluation framework	1. Bibliography: author(s); publication year 2. Setting and aim: country; region; decision-maker; evaluation aim 3. Study design: e.g., decision model 4. Target population/sample demographics and comorbidities: e.g., residence – community-dwelling and/or institutionalised; age; sex; SES; health conditions unrelated to falls risk 5. Type of analysis: e.g., CUA, CEA, CBA, ROI 6. Perspective: e.g., public sector, societal 7. Cost-effectiveness threshold clearly stated 8. Time horizon of analysis/model 9. Discount rates (if time horizon is longer than 1 year)
(B) Falls epidemiology	1. Target population/sample falls risk factors/profile at baseline 2. Fall type: definition; recording method 3. Health consequences of falls: injury type; long-term consequences (e.g., institutionalisation, excess mortality risk) 4. Health utility measurement: acute vs. long-term impact of falls on health utility; comorbidity-related impact on health utility 5. Economic consequences of falls: care resource types; unit costs; all-cause and fall-related costs^a^ 6. Wider/societal consequences of falls: e.g., social isolation from fear of falling; informal caregiver burden; productivity loss of older persons and caregivers
(C) Falls prevention intervention	1. Intervention characteristics: type (e.g., exercise, multifactorial); reach;^b^ primary vs. secondary prevention; main components; staff type; duration, frequency and dose; mutual exclusivity;^c^ comparator(s) 2. Intervention pathway: type (e.g., reactive, proactive, self-referred^d^); recruitment method; falls risk identification method; mutual exclusivity 3. Intervention resource use: e.g., staff labour and training; transport; overheads 4. Intervention costs: variable vs. fixed costs; economies of scale; societal costs (e.g., time opportunity cost, private co-payment) 5. Intervention implementation: uptake rate; adherence rate; sustainability rate 6. Intervention efficacy: risk of bias in estimation; match with incidence metric;^e^ efficacy fall type;^f^ efficacy durability;^g^ wider health benefits; side effects 7. Intervention study characteristics: study design (e.g., RCT, meta-analysis); population/sample characteristics^h^
(D) Decision model features	1. Model type and justification of type 2. Model cycle length and justification of length 3. Methods for adopting a long-term model horizon^i^ 4. Methods for characterising baseline demographics and falls risk of model target population 5. Methods for characterising multiple falls in a year (recurrent falls) 6. Methods for characterising dynamic progression of falls risk factors, long-term consequences of falls and falls prevention intervention need^j^ 7. Methods for characterising dynamic progression in comorbidities and changes in care costs, mortality risks, institutionalisation risks and health utilities 8. Methods for incorporating psychological and sociological variables (e.g., motives for healthy behaviour, community institutions) as determinants of falls risk, falls prevention access and model outcomes 9. Methods for incorporating budget and capacity constraints 10. Methods for reducing structural uncertainty of model prospectively^k^ 11. Model validation methods/results: face; internal; external
(E) Evaluation methods and results	1. Cost-per-unit ratios (e.g., incremental cost per QALY gain) 2. Aggregate health and cost outcomes (e.g., total intervention cost, total QALY gain, total number of falls prevented) 3. Currency: original type/year; conversion to same currency for comparison 4. Handling heterogeneity: subgroup analyses; targeting analyses (under budget or capacity constraint) 5. Handling parameter uncertainty: deterministic sensitivity analysis; probabilistic sensitivity analysis 6. Scenario analyses: testing structural assumptions; scenario suggestions by stakeholders/decision-maker; value of implementation analysis [26] 7. Equity analyses: intervention impact on social inequities in health; estimating efficiency cost or joint equity-efficiency impact of prioritising vulnerable groups (e.g., via distributional cost-effectiveness analysis (DCEA) [27]) 8. Model cross-validity: comparison of results to previous models
(F) Discussions by evaluation authors	1. Discussion on issues of generalisability and policy implementation 2. Discussion on strengths and limitations of evaluation

**Abbreviation:** *CBA* cost–benefit analysis; *CEA* cost-effectiveness analysis, *CUA* cost-utility analysis, *QALY* quality-adjusted life year, *RCT* randomised controlled trial, *ROI* return on investment, *SES* socioeconomic status

^a^Expert guideline on falls prevention economic evaluation recommends that evaluations report all-cause/total healthcare costs in the base case and fall-related costs in sensitivity analysis [22]

^b^Intervention reach refers to the number/proportion of persons in the target population accessing the intervention. It is a function of intervention’s *normative* reach defined by its eligibility criteria and its *implementation* reach determined by implementation level (e.g., uptake rates) within the eligible population

^c^Several intervention types and pathways can be non-mutually exclusive in a setting: e.g., reactive home assessment and modification for fallers discharged from hospitals and self-referred exercise

^d^Reactive pathway is accessed immediately after a fall requiring medical attention. Proactive pathway is accessed via referrals by care professionals in the community. Self-referred pathway is accessed voluntarily by older persons based on community/peer marketing

^e^This only concerns decision models that import falls efficacy evidence from external intervention studies. Main falls incidence metrics are falls risk and falls rate, and their matching efficacy metrics are relative risk (RR) and rate ratio (RaR), respectively. Models should ensure that the external efficacy metric matches the internal falls incidence metric

^f^Like note 5, this again only concerns decision models using external efficacy evidence. The fall type (e.g., hospitalised fall, fall-induced fracture) for the efficacy data should match that for the model incidence

^g^Durability of intervention efficacy should not extend beyond the timespan of the intervention study unless the intervention receipt is sustained [22]

^h^Decision models should ensure that the characteristics of the external intervention study’s target population/sample (e.g., inclusion/exclusion criteria) match those of the model population

^i^Lifetime horizon is recommended by the expert guideline on falls prevention economic evaluation [22]

^j^An example of a method used to characterise the dynamic complexity of falls risk is to incorporate tunnel states in Markov cohort models to capture the secular age-related increase in falls risk [28]

^k^Prospective reduction in structural uncertainty can be achieved through stakeholder engagement and model conceptualisation that precedes model parameterisation [15]

The methodological quality of reviews was further critically appraised narratively. Specifically, the following guidelines and academic papers were used to establish what methodological features and outcomes of falls prevention economic evaluations should be extracted and analysed by the systematic reviews: (a) the expert guideline and checklist on conducting and reporting falls prevention economic evaluation [22]; (b) the review of key methodological challenges to economic evaluation of geriatric public health interventions [23]; (c) the health technology assessment checklist for quality assessment of decision models [24]; and (d) the systematic methodological review of key methodological challenges to public health economic model development [25] and the associated model conceptualisation framework [15]. Table 1 shows the data fields grouped into higher categories. Strengths and limitations stated by the systematic review authors were also noted.

### Commissioning recommendation by this systematic overview

The results and methodological features were extracted from a subset of primary economic evaluations and re-analysed to inform the commissioning recommendations made by the systematic overview. Specifically, data were extracted from general population models (as opposed to models targeting specific patient groups) analysed over lifetime horizons since these are most informative for jurisdiction-level commissioning decisions on falls prevention [22, 29]. Such re-analysis of primary study outcomes is recommended by the Cochrane guideline if this suits the purpose of the systematic overview [20]. Key methodological features of the models that are likely to influence their outcomes are considered while formulating the commissioning recommendations.

## Results

### Systematic overview search results

**Fig. 1:**
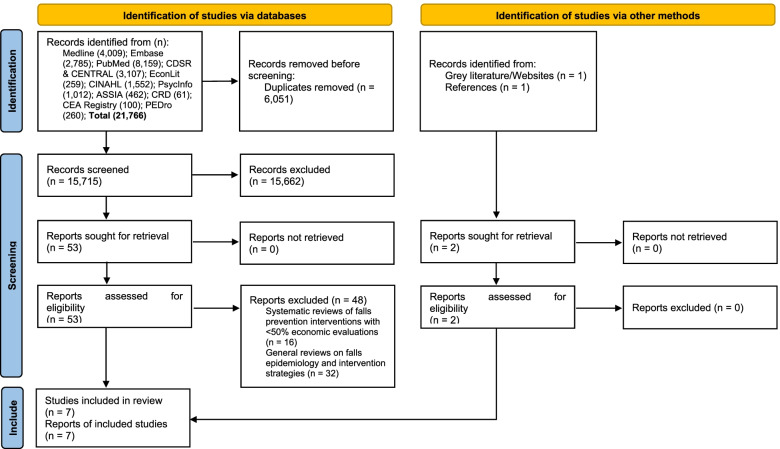
Preferred Reporting Items for Systematic Reviews and Meta-Analyses flow diagram

Figure 1 presents the PRISMA flow diagram: 15,730 titles and abstracts were screened; and 55 full texts screened, from which seven systematic reviews were identified (two from grey literature and references). Table B in Supplementary material lists the 48 studies excluded at the full text screening stage and the reasons for exclusion.

### Methods and findings of previous systematic reviews

Aim, search strategy and search result.

Table 2 summarises the aim, search strategy and search results of previous systematic reviews. The reviews shared the aim of assessing the cost-effectiveness evidence within their targeted intervention area. Two reviews specifically targeted community-based falls prevention interventions [30, 31]; three targeted falls prevention in both community and institutionalised settings [17, 32, 33]; and two targeted a broader range of geriatric public health interventions, more than 50% of which were community-based falls prevention interventions [34, 35]. One only included RCT-based evaluations of falls prevention exercise [33]. Several reviews had further aims of informing: the development of the NICE falls prevention clinical guideline [17]; the development of a new falls prevention decision model [31]; the practice of and research on falls prevention exercise [33]; and the methodologies of subsequent falls prevention economic evaluations [32, 34, 35]. All searches covered at least four academic databases, while three further covered grey literature sites.

**Table 2:** Aim, search strategy and search results of previous systematic reviews of community-based falls prevention economic evaluations

Review	Aim	Search strategy			Search results
		**Coverage period**	**Source**	**Target intervention/setting**	
RCN review [17]	[1] Assess cost-effectiveness of falls prevention interventions (any setting) (2) Inform the NICE clinical guideline on falls prevention for older people	Database inception to April 2003	4 academic databases	Falls prevention interventions in community and extended care	7 evaluations, of which 5 CB falls prevention including 1 model
Davis review [30]	(1) Assess cost-effectiveness of community-based falls prevention interventions	Database inception to July 2008	4 academic databases	Community-based falls prevention interventions	9 evaluations, all CB falls prevention including 3 models
DJ review [34]	(1) Assess cost-effectiveness of public health interventions for older people (any setting) (2) Evaluate methodological features and quality of falls prevention economic evaluations	2000 to July 2015	5 academic databases and 23 grey literature sites	Health promotion and primary prevention interventions (except vaccination) for older people in community and extended care	29 evaluations, of which 22 CB falls prevention including 10 models
PHE review [31]	(1) Assess cost-effectiveness of community-based falls prevention interventions (2) Inform development of falls prevention economic model for English community setting	2003 to December 2016	13 academic databases and 7 grey literature sites	Community-based falls prevention interventions recommended by 2013 NICE guideline (CG161) [16]^b^	26 evaluations, all CB falls prevention including 12 models
Olij review [32]	(1) Assess cost-effectiveness of falls prevention interventions (any setting) (2) Evaluate methodological features and quality of falls prevention economic evaluations	Database inception to May 2017	6 academic databases and Google Scholar	Falls prevention interventions in community and extended care	31 evaluations, of which 28 CB falls prevention including 10 models
Huter review [35]	(1) Evaluate how economic evaluations of public health interventions for older people (any setting) handled key methodological challenges^a^	2000 to March 2018	5 academic databases and 23 grey literature sites	Health promotion and primary prevention interventions (except vaccination) for older people in community and extended care	37 evaluations, of which 25 CB falls prevention including 11 models
Winser review [33]	(1) Assess cost-effectiveness of exercise-based falls prevention interventions (any setting) (2) Evaluate implications for clinical practice and future research on falls prevention exercise dosage	Database inception to February 2019	6 academic databases	Exercise-based falls prevention interventions evaluated by RCTs in community and extended care	12 evaluations, all CB falls prevention including 1 model^c^

**Abbreviation:** *CB* community-based, *DJ* Dubas-Jakobczyk, *NICE* National Institute for Health and Care Excellence, *PHE* Public Health England, *RCN* Royal College of Nursing, *RCT* randomized controlled trial

^a^These are: (i) measurement and valuation of informal caregiving; (ii) accounting for productivity costs (including unpaid work); (iii) accounting for unrelated cost in added life years; and (iv) accounting for wider non-health effects of interventions

^b^This excludes interventions such as vitamin D, hip protectors and cognitive behavioural therapy [31]

^c^One evaluation developed a decision tree model using data from a single falls prevention trial [36]. This was classified as a trial-based evaluation by Winser review

Overall, the reviews identified 44 economic evaluations of community-based falls prevention interventions, of which 21 were decision models. All SVEs except one [37] were evaluations alongside RCTs. Four models used effectiveness evidence from quasi-experimental or observational studies [38–41]; two used efficacy assumptions [42, 43]; the rest relied on efficacy data from individual RCTs or meta-analyses. The recent decade has seen a significant increase in the evaluation number, rising from nine identified by the Davis review [30] in 2008 to 26 identified by the PHE review in 2018 [31]. Table C in Supplementary material provides the reference and characteristics of identified economic evaluations, including their target population, type(s) of analysis, perspective(s), analysis time horizon, intervention(s) and comparator(s). No evaluation was identified by all seven reviews owing to the varying review search strategies (e.g., different coverage periods).

Data fields extracted by systematic reviews.

Table 3 shows the data fields extracted from economic evaluations by previous reviews. There was a marked variation across reviews in the number of data fields extracted, ranging from eight to 33. Data fields for model features were the most limited, restricted to model type and evidence source. No review quantitatively pooled the evaluation outcomes due to significant underlying methodological differences.

**Table 3:** Data fields extracted by previous systematic reviews of community-based falls prevention economic evaluations^1^

Data fields	Systematic reviews						
	RCN [17]	Davis [30]	DJ [34]	PHE [31]	Olij [32]	Huter [35]	Winser [33]
***(A) Setting, population and evaluation framework***							
Author(s) and publication year	˟	˟	˟	˟	˟	˟	˟
Country/region	˟	˟		˟	˟		˟
Study design (e.g., model, RCT)	˟	˟	˟	˟	˟		˟
TP/sample residence	˟	˟	˟	˟	˟		˟
TP/sample age and sex	˟	˟	˟	˟	˟		˟
Type of analysis (e.g., CUA)		˟	˟	˟	˟	˟	˟
Perspective (e.g., societal)		˟	˟	˟	˟	˟	˟
Time horizon/Follow-up period		˟	˟	˟	˟		˟
Discount rates		˟		˟	˟		˟
*Number of fields*	5	9	7	9	9	3	9
***(B) Falls epidemiology***							
TP/sample falls risk factor(s)		˟		˟	˟		˟
Baseline falls risk estimates					˟		
Main health event (e.g., fall type)	˟	˟	˟	˟	˟		˟
Health utility instrument					˟		˟
Wider (e.g., non-health) outcomes						˟	
Health and social care consequence types		a	˟		˟		˟
Societal consequence types		a	˟		˟	˟	˟
All-cause/comorbidity costs		a				˟	
Cost measurement method in RCT					˟		
*Number of fields*	1	5	3	2	7	3	5
***(C) Falls prevention intervention***							
Intervention type	˟	˟	˟	˟	˟		˟
Primary vs. secondary prevention				˟			
Intervention components		˟		˟	˟		˟
Intervention duration		˟		˟			˟
Exercise intervention dosage							˟
Professional staff involved		˟		˟			˟
Comparator		˟	˟	˟	˟		˟
Participant recruitment method/setting		˟		˟			˟
Falls risk identification method				˟			
Intervention resource use		˟		˟			˟
Intervention cost		b	˟	˟			˟
Societal intervention resource/cost						˟	˟
Intervention fall-related efficacy		˟		˟			˟
Intervention study sample size		˟	˟	˟	˟		˟
*Number of fields*	1	10	4	12	4	1	12
***(D) Decision model features***							
Model type			˟	˟	˟		
Model data sources			˟	˟			
Characterising baseline falls risk estimates					˟		
*Number of fields*	0	0	2	2	2	0	0
***(E) Evaluation methods and results***							
Cost-per-unit ratio (e.g., ICER)	˟	˟	˟	˟	˟		˟
Aggregate cost and health outcomes^2^		˟		˟			˟
Original currency type		˟	˟				˟
Converted results into same currency		˟	˟		˟		
Subgroup/targeting methods/results		˟		˟	˟		˟
Handling parameter uncertainty^3^		˟		˟	˟		˟
Scenario analysis methods/results^4^				˟			
Equity analysis methods/results				˟			
*Number of fields*	1	6	3	6	4	0	5
***(F) Discussions by evaluation authors***							
Generalisability and policy implementation			˟	˟			
Strengths and limitations			˟	˟		˟	
*Number of fields*	0	0	2	2	0	1	0
*Total number of fields*	8	30	21	33	26	8	31

**Abbreviation:** *CUA* cost-utility analysis, *DJ* Dubas-Jakobczyk, *ICER* incremental cost-effectiveness ratio, *PHE* Public Health England, *RCN* Royal College of Nursing, *RCT* randomized controlled trial, *TP* target population

^1^This table does not account for data fields extracted by reviews for applying a quality assessment checklist

^2^Includes outcomes such as total intervention cost and total number of falls prevented

^3^ncludes one-/two-way deterministic sensitivity analysis and probabilistic sensitivity analysis

^4^Analysis of alternative modelling assumptions: e.g., whether fear of falling exerts a health utility decrement

^a^Distinguished between fall-related and all-cause care cost and reported detailed list: emergency department; hospitalization; outpatient visit; GP visit; district nurse visit; home care; equipment; meal-on-wheel; day care centre; residential care; nursing home; patient and caregiver’s cost (out-of-pocket expenditure, time cost)

^b^Reported detailed list of intervention resources for costing: recruitment; marketing; printing; development; administration; overheads; staff labour; staff transport; training; equipment; home modification; specialist service (e.g., cataract operation); comparator intervention resource/cost

Quality assessment of economic evaluations by systematic reviews.

All reviews except RCN (which mentioned applying the Drummond checklist [18] but did not report the scores) applied a checklist to assess the reporting and methodological quality of their included studies. In total, four checklists were applied, all of them generic (i.e., all disease areas) and all-design (i.e., SVEs and models). Table D in Supplementary material lists the items of the checklists used, and Table E shows the quantitative checklist scores given to individual economic evaluations by the reviews. The scores were converted to percentage to ease comparison.

Thirteen out of 24 SVEs and 11 out of 21 models received scores from multiple reviews. The last column of Table E shows the standard deviation (SD) of scores per evaluation. The SD varied markedly between evaluations, ranging from 0.9 to 45.0. The average checklist scores were also calculated for each review by study design. By comparing an individual evaluation’s score against the average, its relative quality ranking (above or below average) within each review could be determined. There were hence potential differences in how reviews perceived the relative quality of their included evaluations based on the checklist scores (though the relative rankings would also depend on what evaluations are included). For example, Hektoen (2009) received the Drummond checklist score of 90.0% in the DJ review and was above the review average for models (70.9%); but it received NICE checklist score of 26.3% in the PHE review which was markedly below the review average for models (59.6%).

In addition to checklists, the DJ review narratively synthesised limitations of included studies around the following methodological themes: identifying and measuring costs and benefits; uncertainty over input variables; short time horizon; problems with sample (e.g., low participation); and problems with generalizability. The PHE review noted the main limitations of evaluations as perceived by the evaluation authors or reviewers but did not group them by themes. The Huter review narratively synthesised how evaluations handled the challenges of societal analysis, namely the incorporation of: (1) informal caregiving cost; (2) productivity cost; (3) unrelated cost in added life years; and (4) wider non-health effects. It was found that these challenges were handled in few evaluations; and when handled, were done using very heterogenous methods.

Commissioning and research recommendations in systematic reviews.

Table 4 summarises the commissioning and research recommendations made by previous reviews.

**Table 4:** Commissioning recommendations and research implications from previous systematic reviews of community-based falls prevention economic evaluations

Review	Commissioning recommendations	Research recommendations/implications
RCN [17]	• No commissioning recommendation based on systematic review results	•Development of a de novo decision model to inform NICE clinical guideline [17]
Davis [30]	• “We conclude that single interventions (such as the Otago Exercise Programme) targeted at high-risk groups can prevent the greatest number of falls at the lowest incremental costs.” (p. 89)	• “We recommend that future economic evaluations be guided in part by the checklists available for assessing economic evaluations.” (p. 88) •Development of guideline and checklist for falls prevention economic evaluations [22]
DJ [34]	•Cost-effective/cost-saving interventions in ‘Good’ quality studies: resistance exercise; Otago exercise; Tai Chi; citywide non-pharmaceutical multifactorial programme • “The existing studies are characterized by huge differences in the methods applied as well as overall quality which limits the comparability and generalizability of the results.” (p. 670)	• “There is a need for… methods adjusted to particular character of health promotion and primary prevention strategies for older population.” (p. 670)
PHE [31]	•Exercise interventions (p. 39–40): Tai Chi is consistently most cost-effective for mobile older persons; group exercise for women aged 70 + cost-effective; Otago home exercise may be cost-saving with high adherence; other home exercises are not cost-effective •Multifactorial interventions (p. 40): paramedic-implemented protocol that followed NICE guideline was cost-saving and is generalizable to English setting; risk assessment without treatments not cost-effective •HAM likely cost-effective but current evidence not generalizable to English setting (p. 40–41) •Medication review likely cost-effective (p. 41)	•Falls prevention economic model should carefully consider whether the intervention being modelled is appropriate for English setting and given target population (p. 44) •Development of a de novo decision model to inform commissioning of falls prevention by CCGs/local authorities [44]
Olij [32]	• “Home assessment programs were most cost-effective type of program [based on CUA] for community-dwelling older adults.” (p. 2197) • “Multifactorial programs and other [e.g., exercise] programs were less favourable [based on CUA].” (p. 2202) • “Older populations reported more favourable ICERs… [but] it is not possible to draw firm conclusions about age differences.” (p. 2202) • “Methodological differences between studies hampered direct comparison of the cost-effectiveness of program types.” (p. 2197)	• “Future economic evaluations of falls prevention should be designed, conducted, and reported in accordance with current guidelines for economic evaluations to increase comparability.” (p. 2202) • “Future studies should clearly report whether they target high-risk, low-risk, or mixed populations because the baseline fall risk is an important determinant of cost-effectiveness.” (p. 2202) •Models should directly compare different falls intervention types (p. 2202)
Huter [35]	• “A comparison of results of different economic evaluations, even of similar interventions, has to be carried out with great caution.” (p. 8) • “A comparison of the cost-effectiveness results with… other age groups is not possible and therefore not advisable.” (p. 9)	• “Disregarding [the four features^a^] could implicitly lead to a discrimination of health promotion and disease prevention against older people.” (p. 9) • “More research is necessary on the different approaches for [the four features’] inclusion and on their respective effects on the outcomes.” (p. 9)
Winser [33]	• “A tailored exercise program including strengthening of lower extremities, balance training, cardiovascular exercise, stretching and functional training of moderate intensity performed twice per week with each session lasting 60 min for 6 or more months delivered in groups of 3 to 8 participants [by PT or nurse trained by PT] with home-based follow-up appears to be cost-effective in preventing falls in older people.” (p. 69) • “Exercise-only programs were more cost-effective than multifactorial falls prevention programs.” But “there were not enough studies of each to draw firm conclusions.” (p. 75, 78)	• “We recommend future studies to test the benefits of adding scheduled walking to the falls prevention exercise protocol.” (p. 76) • “Research is needed to evaluate the efficacy of [group-based learning and home-based practice] programs, in particular in comparison to other programs that may require more resources.” (p. 76) • “Further research is needed… in developing and underdeveloped countries.” (p. 69) • “Future research is needed to systematically compare [exercise-only and multifactorial programs].” (p. 78)

**Abbreviation:** *CCG* clinical commissioning group, *CUA* cost-utility analysis, *HAM* home assessment and modification, *NICE* National Institute for Health and Care Excellence, *PT* physiotherapist

^a^These are: (i) measurement and valuation of informal caregiving; (ii) accounting for productivity costs (including unpaid work); (iii) accounting for unrelated cost in added life years; and (iv) accounting for wider non-health effects of interventions

Scarce cost-effectiveness evidence from the UK setting prevented the RCN review from making commissioning recommendations. The Davis review recommended single-component Otago home exercise based on the most favourable cost-per-unit ratio. The DJ review reported three exercise interventions and a citywide multifactorial intervention that produced the lowest cost-per-unit ratios from ‘Good’ quality evaluations (those that received 90–100% Drummond checklist score). The PHE review based recommendations by intervention type on cost-per-unit ratios. The Olij review recommended HAM over exercise and multifactorial interventions for community-dwelling older persons based on incremental cost per QALY ratios under CUA. The Winser review listed the characteristics of an ideal exercise intervention based on those of interventions that yielded favourable cost-per-unit ratios. It also found that single-component exercises produced more favourable ratios than exercises within multifactorial interventions but called for further direct comparisons.

For research implications, the RCN and PHE reviews determined that a de novo model is required to assist commissioning due to lack of current evidence. The Davis and Olij reviews recommended that future evaluations follow a validated guideline or checklist for economic evaluations. The Davis review later informed the development of the expert guideline/checklist for falls prevention economic evaluations [22]. The Huter review stressed that future evaluations should incorporate the four methodological challenges associated with societal analyses (given above) to counteract the indirect bias of economic evaluations against older age groups (e.g., due to reduced scope of QALY gain). It should nevertheless be noted that inclusion of productivity costs would favour economically active/younger populations (see the results of Johansson (2008) [40] in Table 5 below).

**Table 5:** Characteristics and results of lifetime modelling studies identified by included systematic reviews

Study	Analysis; Perspective	Target population	Falls epidemiology	Intervention features	Evaluation results^a^	Methodological caveats
Church (2012) [46]	CEA/CUA; Public healthcare	Australian CD adults aged 65 +	***Data source*:** literature; expert opinion ***Fall type*:** non-MA fall; MA fall; hip fracture; fear of falling; fatal fall ***Economic*:** ED; inpatient; rehab.; LTC	***Type*:** (i) General – Group exercise; Home exercise; Tai Chi; Multi-component int.; Multifactorial int.; Multifactorial risk assessment; (ii) High-risk – Group exercise; HAM; Multifactorial int.; (iii) Specific – Expedited cataract surgery; Cardiac pacing; Psychotropics withdrawal ***Comparator*:** UC; Cross comparisons ***Resource/cost*:** Per-participant cost only ***Implementation*:** 1-year maintenance^b^	***Ratios*:** (i) General – Tai Chi ICER £27,734 per QALY vs. UC; other interventions dominated; (ii) High-risk – Group exercise ICER £31,957 per QALY vs. UC; HAM ICER £36,298 per QALY vs. UC; Multifactorial int. dominated; (iii) Specific – Expedited cataract surgery dominated UC and other interventions. ***Aggregate*:** reports incremental cost, no. of falls avoided and QALY gain per intervention, but all interventions have same reach^c^ (including those targeting high-risk and specific subgroups), and hence cannot compare aggregate impacts. ***Parameter uncertainty*:** CEAC; one-way sensitivity analyses on ICER ***Scenario analyses*:** No fear of falling had substantial impact	Unclear falls risk progression;^d^ Recurrent falls not characterised;^e^ Unclear intervention reach;^c^ Unclear how high-risk subgroup identified; Mismatch between falls incidence and efficacy metrics; No fixed int. cost; No capacity constraints
Farag (2015) [42]	CUA; Public healthcare	Australian CD adults aged 65 + without prior fall	***Data source*:** literature ***Fall type*:** non-MA fall; MA fall; fatal fall ***Economic*:** ED; inpatient; LTC	***Type*:** Non-specific falls prevention int. with relative risk of 0.75 and per-participant cost of £420 ***Comparator*:** UC ***Resource/cost*:** Per-participant cost only ***Implementation*:** 50% uptake in base case; maintenance not stated	***Ratios*:** ICER of £17,320 per QALY vs. UC ***Aggregate*:** incremental cost and QALY gain outcomes per person can be scaled up but unclear to what extent. ***Parameter uncertainty*:** CEAC; 57% probability of being cost-effective at AUS$50,000 threshold; one-way sensitivity analyses on ICER ***Scenario analyses*:** e.g., variation in uptake rate had little impact on ICER	Unclear falls risk progression;^d^ Recurrent falls not characterised;^e^ No discounting; No fixed int. cost; No capacity constraints
Johansson (2008) [40]	CUA; Societal	Swedish CD adults aged 65 + (n = 5,500)	***Data source*:** int. study ***Fall type*:** hip fracture; excess mortality ***Economic*:** primary care; inpatient; outpatient; pharma.; LTC; informal care; productivity loss; comorbidity net consumption cost (in scenario)	***Type*:** Multifactorial and environmental int.^f^ ***Comparator*:** UC ***Resource/cost*:** Reports total int. cost; Includes cost of stakeholder involvement, volunteer labour and time opportunity cost ***Implementation*:** not stated	***Ratio*:** intervention had higher health gain and lower cost (dominated) comparator ***Aggregate*:** total int. cost of £640,918; total costs savings of £647,970; total QALY gain of 35.16 ***Parameter uncertainty*:** Scatter plot ***Scenario analyses*:** Intervention dominated UC for age groups 65–79 and 80 + by sex. Scenarios that made intervention no longer dominant – doubled fracture risk; lower treatment cost of fracture; inclusion of comorbidity net consumption cost;^g^ higher discount rate; no health/cost consequences of fracture beyond 1^st^ year; 25% rise in int. cost	Unclear falls risk progression;^d^ Quasi-experimental study for effectiveness evidence; No tiered threshold for evaluating societal outcomes;^h^ Internal and external validation conducted
OMAS (2008) [47]	CEA/ROI; Public healthcare	Canadian CD adults aged 65 +	***Data source*:** routine data analysis ***Fall type*:** MA fall; excess mortality ***Economic*:** ED; inpatient; rehab.; LTC	***Type*:** Exercise; HAM; Vitamin D & calcium; Psychotropics withdrawal; Gait stabilizing device; Eligibility for each intervention defined by relevant falls risk factor ***Comparator*:** UC ***Resource/cost*:** Per-participant cost only ***Implementation*:** Unique uptake and adherence rates for each intervention; Permanent maintenance for 1^st^ year adherers	***Ratio*:** All interventions dominated UC under CEA for men and women ***Aggregate*:** Reports net cost saving per person which can be scaled up to total for each intervention subgroup at regional level ***Parameter uncertainty*:** No analysis ***Scenario analyses*:** No analysis	Unclear falls risk progression;^d^ Recurrent falls not characterised;^e^ Mismatch between intervention need and falls risk;^i^ Parameter uncertainty not assessed
Pega (2016) [48]	CUA; Public healthcare	New Zealand CD adults aged 65 +	***Data source*:** routine data analysis ***Fall type*:** indoor MA fall; fatal fall ***Economic*:** primary care; pharma.; rehab.; inpatient; comorbidity healthcare cost^j^	***Type*:** HAM ***Comparator*:** UC ***Resource/cost*:** Per-participant cost only ***Implementation*:** One-off HAM yields lifetime efficacy (10 years in scenario)	***Ratio*:** HAM had ICER of £5,123 per QALY vs. UC in base case ***Aggregate*:** For base case, total int. cost was £82.5 million, total net cost vs. UC £62.6 million and total QALY gain 34,000. ***Parameter uncertainty*:** 95% uncertainty interval for ICER between below zero to £11,385 per QALY; one-way sensitivity analyses ***Scenario analyses*:** For secondary prevention scenario,^k^ ICER was £1,139 per QALY, total int. cost £10.2 million, total net cost vs. UC, £3.5 million, and total QALY gain 20,100. Targeting those aged 75 + produced ICER of £8,956 per QALY, total net cost vs. UC £31.1 million, and total QALY gain 8,750. Subgroup analyses showed higher ICERs for Maori and men. Equity analyses showed that the higher ICERs can be mainly attributed to shorter life expectancies of Maori and men	Unclear falls risk progression;^d^ Recurrent falls not characterised;^e^ Routine data lacks individual identifier;^l^ No background transition in health utilities; No fixed int. cost; No capacity constraints; No scenario estimating efficiency cost^m^

**Abbreviation:** *CEA* cost-effectiveness analysis, *CD* community-dwelling, *CUA* cost-utility analysis, *ED* emergency department, *HAM* home assessment and modification, *ICER* incremental cost-effectiveness ratio, *int* intervention, *LTC* long-term care admission, *MA fall* fall requiring medical attention, *OMAS* Ontario Medical Advisory Secretariat, *pharma* pharmaceuticals, *QALY* quality-adjusted life year, *rehab* rehabilitation, *ROI* return on investment, *UC* usual care

^a^All monetary units are converted to £ in year 2021 using the average consumer price index (CPI) between the original year of reported currency to 2019 (most recent year for CPI data) in the country of study and purchasing power parity (PPP) rate between the original currency and £ in year 2020 (most recent PPP data)

^b^Maintenance refers to the duration of eligible persons receiving the intervention. Intervention effectiveness is a function of efficacy durability and maintenance period

^c^Intervention reach refers to the number/proportion of persons receiving the intervention. It is a function of intervention’s *normative* reach defined by its eligibility criteria and targeting strategy and its *implementation* reach determined by the level of implementation (e.g., uptake, adherence, sustainability) within the eligible population

^d^Specifically, the study does not mention how falls risk progressed with age in the absence of falls incidence (which has a separate model state). Markov model should incorporate tunnel states to allow for secular risk progression, but this is not stated or graphically illustrated

^e^Markov models with 1-year cycles should assign the number of falls to individual fallers who experience at least one fall in a given 1-year cycle or include a separate model state for being a recurrent faller. Not incorporating recurrent falls would underestimate the health burden of falls

^f^Multifactorial intervention in this study included tailored education, group balance exercises, Tai Chi, other physical activities and HAM. Environmental intervention included neighbourhood hazard removal and housing reconstruction

^g^The study incorporated cost of added life-years which was estimated as the consumption minus production level (i.e., net consumption) that varied by age group. The outcome changed from dominance to ICER of £16,980 per QALY

^h^Societal costs incur different opportunity cost to public sector costs. The cost-effectiveness threshold should be tiered or weighted to capture the differing opportunity costs across sectors

^i^ he study estimated the proportion of target population who would be eligible for each of the interventions according to the prevalence of falls risk factors that defined eligibility: exercise for mobile older persons without disability (65.8%); HAM for frail older persons with disability (16.9%); vitamin D for women with fracture risk factors (52.9% of female); psychotropics withdrawal for psychotropic users (11.8%); and gait stabilizers for mobile seniors without disability (65.8%). However, the falls risk in the model was determined only by age, sex and MA falls history. Hence, different intervention subgroups had similar falls risk despite contrasting risk factor profiles

^j^The study incorporated healthcare cost of added life-years and cost of dying (healthcare cost in last 6 months) which varied by age group and sex

^k^This scenario involved HAM targeted at subgroup with history of MA fall. This subgroup comprised 10% of target population

^l^Without individual identifiers, multiple falls experienced by the same person are counted as multiple fallers

^m^The study evaluated counterfactual scenarios where Maori/men had equal life expectancy as non-Maori/women and found that subgroup ICERs became similar. This, however, does not estimate the efficiency cost incurred if Maori/men are prioritised for intervention under the factual circumstance of lower life expectancy

### Critical appraisal of previous systematic review methodology

Table F in Supplementary materials shows the results of applying the 16-item AMSTAR 2 checklist to the systematic reviews. No review conducted meta-analysis due to methodological heterogeneity among the included evaluations. Therefore, the maximum potential number of ‘Yes’ (i.e., full adherence to item criterion) or ‘Partial Yes’ was 13 since items 11, 12 and 15 only concerned meta-analyses. The RCN review had the lowest number of ‘Yes’ at two, followed by the Davis review at seven. The five later reviews had nine or 10 ‘Yes’, suggesting that the review methods have improved over time. The most prevalent issue was the omission of a list of excluded studies (item 7), with only two reviews providing it. The second most prevalent issue was the lack of consideration of methodological quality of evaluations when discussing and formulating review conclusions (item 13). The Olij review, for example, applied the CHEC methodological quality checklist but did not discuss the checklist scores when comparing the ICERs of evaluations.

Limitations acknowledged by the review authors included: limited search coverage [31–34]; lack of quantitative meta-analysis [31, 33]; non-assessment of publication bias [31, 32]; and limited assessment of the quality of underlying clinical studies [31, 32].

Two further limitations of systematic reviews can be noted by this systematic overview:

1. The limited range of methodological features extracted from studies, particularly models; and
2. The limited range of evaluation outcomes extracted to inform commissioning.

The first limitation is made clear by comparing Tables 1 and 3. There was a marked difference between what data fields could or should have been extracted by systematic reviews according to expert guidelines and literature [15, 22–25] (Table 1) and those extracted (Table 3). Decision model features were the most neglected category. One particularly important (yet neglected) set of modelling features are methods for characterising the dynamic progression in falls risk and falls prevention intervention need. An individual’s falls risk profile encompasses multiple interacting risk factors – including age, falls history, physical function (e.g., gait and balance) and cognitive function [16] – which are all highly dynamic; and changes to the falls risk profile would then entail changes to intervention need and eligibility. As far as time and resources permit, systematic reviews should account for how such features were modelled, including the data sources and parameters used and structural assumptions made. Insofar as models – and particularly population-level long-horizon models – provide the most relevant information to commissioners, the reviews’ limited focus on the modelling features reduces their capacity to inform not only the commissioning decisions but also the conceptualisation of future falls prevention economic models.

The second limitation concerns the way in which reviews’ commissioning recommendations were based chiefly on cost-per-unit ratios without considering aggregate outcomes. For example, the Davis review recommended the Otago home exercise for population aged 80 + based on a single SVE result that the intervention produced a net cost saving [45]. Yet another evaluation in the review reported a similar cost saving from a citywide intersectoral intervention over a five-year horizon [39]. Even with comparable cost-per-unit ratios, consideration of aggregate impact would favour the citywide intervention. The cost-per-unit ratio also provides little information on the coverage of priority subgroups within the target population. For example, the Olij and Winser reviews recommended HAM and exercise, respectively, over multifactorial interventions based on comparisons of cost-per-unit ratios alone. Yet multifactorial interventions may achieve greater coverage of the most vulnerable patient groups (e.g., those contraindicated for exercise) and hence may be preferred by commissioners who aim to prioritise the care of such groups. Alternatively, HAM/exercise and multifactorial intervention may be commissioned as non-mutually exclusive options, with the more cost-effective option subsidising the lesser. The cost-per-unit ratios estimated in the absence of any capacity constraint should also be interpreted with caution since they would rise quickly once the intervention scale reaches the capacity limit.

### Commissioning recommendation by this systematic overview

Assuming that decision-makers overseeing a health jurisdiction (e.g., at city, state or national level) would prefer general population, lifetime evidence to capture the full health and economic impacts of falls for the whole jurisdiction rather than specific patient groups [22, 29], Table 5 summarises the characteristics and results of five general population, lifetime models that were identified by the previous systematic reviews. Two principles are maintained in interpreting the model results: (I) attention is paid to methodological features that may influence the outcomes or the applicability of the outcomes to the decision-making setting (see category (D) in Table 1); and (II) recommendation is based on a wide range of reported outcomes, not cost-per-unit ratio alone (see category (E) in Table 1

Concerning principle (I), two salient features emerge from Table 5. First, as shown in the falls epidemiology column, there is significant between-study variation in the fall-related health and economic consequences incorporated and in the data sources used to characterise falls risk. Hence, the decision-maker preference over the range of fall-related health and economic consequences would influence the results’ applicability. Secondly, each evaluation has several methodological caveats (see last column) that may affect the credibility of model results. For example, all five studies developed Markov cohort models but mentioned no tunnel states to account for the secular age-related increase in falls risk, which would bias the result against those who are younger at baseline (and against early prevention). Only Johansson (2008) assessed the model’s external validity. The decision-maker should consider these methodological shortcomings when using the model evidence.

Four models that conducted CUA produced cost-per-unit ratios for at least one intervention relative to usual care that can be deemed cost-effective under the cost-effectiveness threshold of £30,000 per QALY gain (i.e., the NICE health technology assessment threshold [49]). In the order of increasing ICER values, the results were:

- Combined multifactorial and environmental intervention in Johansson (2008) with QALY gain and lower cost relative to usual care [40];
- HAM in Pega (2016) with ICER of £5,123 per QALY if implemented for the whole population and £1,139 if targeted at those with history of falls requiring medical attention (MA falls) [48];
- A non-specific intervention in Farag (2015) of £420 per-participant cost and 25% reduction in falls risk with ICER of £17,320 per QALY [42];
- Tai Chi in Church (2012) with ICER of £27,734 per QALY [46].

Given the favourable ratios, a key decisional factor under principle (II) is the population reach of each intervention that determines its aggregate impact, as well as any budget and capacity constraints of the decision-maker. For example, it may be the case that Tai Chi enjoys a substantially greater uptake rate than HAM in the decision-making setting (perhaps due to high prevalence of rented accommodations which makes home modification difficult [48]). In this case, Tai Chi would generate greater aggregate gain (measured by incremental net monetary benefit that incorporates QALY gain and net costs) than HAM despite its higher ICER. But if there are significant budget or capacity constraints such that the wide Tai Chi uptake cannot be realised, then HAM would be preferred since it delivers more health gain per monetary unit of investment. A similar comparison should be made between universal provision of HAM and its targeted provision in Pega (2016). The targeted approach generates lower ICER but generates lower total QALY gain than universal provision: 20,100 QALYs at £3.5 million total net cost vs. 34,000 QALYs at £62.6 million total net cost. The additional 13,900 QALYs from universal provision is of greater value than the £59.1 million additional net cost if the cost-effectiveness threshold is greater than £4,252 per QALY. Thus, the targeted approach should be pursued only if there are budget/capacity constraints (or an equity objective; see below) that preclude the universal provision.

The combined multifactorial and environmental intervention in Johansson (2008) potentially has the greatest reach since it addresses community-wide environmental risk factors (independently of demand by older people) as well as individually tailored treatments including Tai Chi and HAM. However, the model is based on evidence from a quasi-experimental study in a small community of 5,500 older people, and there is no supplementary evidence that it can be successfully implemented in other communities. Hence, the decision-maker should first consult local stakeholders to determine whether the intervention in Johansson (2008) can be scaled up within the budget and capacity constraints. Whether older people’s productivity is considered in the evaluation is another decisional factor since the outcome changes from dominance to ICER of £16,890 per QALY if net consumption cost in added life-years is included.

OMAS (2008) was the only model to conduct CEA for five single-component interventions relative to usual care: exercise, HAM, vitamin D & calcium, psychotropics withdrawal, and gait stabilising device [47]. All interventions reduced the number of MA falls and the net healthcare cost, thus dominating usual care. Gait stabilising device produced the highest reduction in MA falls and net cost *and* had the greatest population reach (65.8%) and hence should be the preferred option. However, there were two main methodological caveats. First, no assessment of parameter uncertainty was conducted despite the paucity of evidence for several model parameters (e.g., only one trial was available for efficacy of gait stabilising device). Secondly, the population reaches of interventions were not based on the characteristics of the simulated model population but imposed exogenously. For example, gait stabilising device was eligible only for mobile seniors without disability, and according to an external survey, this group comprised 65.8% of the general geriatric population. The study then simply assumed that 65.8% of health gains and costs accrue to this intervention subgroup. But the simulated model population were defined by age, sex and MA falls history, not mobility or disability, and hence the true reach of gait stabilising device is unknown. These caveats reduced the credibility of the reported results.

Another key decisional factor under principle (II) is equity consideration beyond cost-effectiveness. Here, only Pega (2016) disaggregated the evaluation results into social subgroups: female vs. male; and non-Maori majority vs. Maori ethnic minority in New Zealand. Male and Maori subgroups had higher ICERs than their respective counterparts, and gained less QALYs per person (e.g., 0.046 for Maori vs. 0.060 for non-Maori). Hence, universal HAM provision *worsens* the health inequity between Maori and non-Maori (the decision-maker may similarly see the health inequality between men and women as unfair). Though the specific ethnic divide is unique to New Zealand, the decision-maker may choose to generalise this case to predict the distribution of HAM impact across locally relevant gradient in social marginalisation. Having done so, commissioning can consider HAM strategies that do not exacerbate the existing health inequity – e.g., targeting the socially marginalised group – even at the expense of reduced cost-effectiveness. It should also consider using an evaluation method that estimates the joint equity-efficiency impact given the decision-makers’ level of inequity aversion [27]. Similar considerations are warranted for other cost-effective interventions, but there are insufficient subgroup results from other models to enable this.

Pega (2016) also provides an insight into the underlying cause of inequitable subgroup impacts. A scenario analysis was conducted wherein the Maori subgroup is assigned the longer life expectancy of the non-Maori subgroup, and it was found that Maori’s QALY gain becomes *higher* than that of non-Maori (0.071 vs. 0.060) and the ICERs become similar. Hence, the inequitable impact can be attributed mainly to the life expectancy differential between ethnic subgroups – though other potential causes of inequitable impact (e.g., lower intervention uptake or efficacy among the Maori) cannot be investigated due to homogenous parameter assumptions across ethnic subgroups. This suggests that falls prevention commissioning should be complemented by upstream interventions at earlier life stages to correct the life expectancy differential that emerges at age 65.

It should also be noted that life expectancy differential exists between age subgroups. Indeed, Pega (2016) estimated an ICER of £8,956 per QALY gained relative to UC for those aged 75 + which was higher than the ICER of £5,123 per QALY gained for those aged 65 + , despite the former’s higher falls burden (and higher potential gain from falls prevention). But the ICER difference should not motivate the targeting of the younger age groups, particularly since both ICERs were comfortably below the £30,000 per QALY gained threshold. Such targeting would also go against the principles of healthcare systems such as those of the NHS and NICE [50, 51]. Younger subgroups can instead cross-subsidise their older peers.

Overall, the commissioning recommendations of this systematic overview are as follows:

1. There is some evidence that combined multifactorial and environmental intervention, HAM and Tai Chi are cost-effective over the lifetime for general geriatric populations aged 65 + .
2. The decision-maker should investigate the feasible reaches of the above interventions in the local setting within the budget and capacity constraints. The reaches concern the intervention’s population coverage and its sustainability over time. Commissioning of additional implementation support (e.g., peer motivators) can also be considered.
3. There is some evidence that national provision of HAM exacerbates the existing health inequity across social subgroups, and this may generalise to the other two interventions. The decision-maker could consider targeting the intervention at socially marginalised groups or a universal provision supplemented by additional implementation support for the marginalised groups. Upstream interventions at early life stages can also supplement falls prevention to reduce the life expectancy differential between subgroups.
4. There are methodological caveats that may significantly influence the model outcomes. The decision-maker could consider commissioning the development of a de novo general population, lifetime model that addresses the main methodological challenges, such as the dynamic complexity in falls risk profile and the psychological and sociological factors that influence the intervention reach and hence its aggregate impact.

## Discussion

This systematic overview identified seven systematic reviews containing 44 falls prevention intervention economic evaluations for older people living in community. The number of data fields extracted from studies differed markedly across reviews, ranging from eight to 33. Four checklists were applied by reviews, while narrative quality assessment was conducted at varying levels of detail and topic range. Commissioning recommendations were based primarily on cost-per-unit ratios. Research recommendations ranged from a call for greater adherence to pre-established guidelines for economic evaluations to development of de novo decision models. The systematic overview made its own commissioning recommendations and critically appraised the methods of previous reviews, particularly regarding the extraction of methodological features and the synthesis of evaluation outcomes.

Application of the AMSTAR 2 checklist showed some evidence of an improvement in systematic review methods, from full adherence to only two checklist items in the RCN review in 2005 to nine or 10 items in the five reviews published in 2017 or later. The low performance of the RCN review is of particular concern given that it informed the development of NICE CG161. Certain aspects of AMSTAR 2 were mainly relevant to systematic reviews of intervention effectiveness studies rather than of economic evaluations. Thus, the checklist items 11, 12 and 15 concerning meta-analysis were less relevant to the reviews that did not pool outcomes due to the underlying methodological heterogeneity in economic evaluations. Moreover, items 2, 9 and 13 concerning risk of bias assessment had to be expanded to address the reviews’ broader methodological quality assessment of evaluations. The question in item 8 of whether the reviews described the evaluations in ‘adequate detail’ required background knowledge of the important features of falls prevention economic evaluations: i.e., the data fields in Table 1 informed by the broader literature on falls prevention and public health economic evaluation and modelling [15, 22–25]. A previous overview in community pharmacy economic evaluation similarly combined the AMSTAR 2 checklist with methodological criteria drawn from the broader literature [52]. Accounting for the *volume* of extracted detail in item 8 nevertheless does not capture the *type* of detail (e.g., dynamic model features, non-health outcomes, equity analyses). Hence narrative synthesis should supplement the checklist application for the appraisal of systematic reviews.

A noticeable finding of this overview was that the extraction and analysis of decision model features by previous systematic reviews was highly limited, although this was intentional in a couple of cases: Huter review focused on a pre-specified list of methodological challenges, while Winser review focused on RCT-based SVEs. The limited appraisal greatly compromises the ability of systematic reviews to inform decision-making at the population level over a time horizon long enough to capture all relevant costs and consequences of a preventive intervention [29, 49]. According to the systematic methodological review that informed the data fields in Table 1, the key methodological challenges within public health economic model development include: (I) incorporating wider costs and effects; (II) considering dynamic complexity (e.g., long-term progression of falls risk factors); (III) incorporating psychological and sociological factors (e.g., those affecting intervention uptake/adherence); and (IV) considering social determinants of health and conducting equity analyses [25]. The Huter review covered only (I), while the PHE review only (IV). Future systematic reviews of public health economic models should endeavour to cover as many of these aspects as possible. This would help judge the structural validity and credibility of included models before they inform commissioning decisions and/or conceptualisation of de novo falls prevention economic models. It would also inform additional commissioning strategies that could supplement falls prevention, such as upstream interventions to address the underlying social disadvantages resulting in inequitable impact of falls prevention [53], and implementation strategies to increase falls prevention uptake [26, 54–57].

A possible contributory factor to the neglect of decision model features is the nature of checklists used by previous systematic reviews to assess the reporting and methodological quality of their identified economic evaluations. All four checklists used by the reviews were designed for all disease areas and for all study designs. Though reviewers are not confined to extracting only the checklist items, the use of a generic, all-design checklist would likely reduce the effort spent in identifying how evaluations captured the disease- and modelling-specific features. Thus, using the fall-specific (but all-design) checklist designed by falls prevention experts [22] may improve the attention paid to features of falls epidemiology and falls prevention intervention by future systematic reviews, while using the model-specific (but generic) HTA checklist [24] may similarly improve the attention on modelling features. However, any quantitative checklist is likely too limited to serve as the main methodological assessment tool. Specifically, its use of binary/ordinal item scores, followed by aggregation to a single index, conceals the highly idiosyncratic nature of methodological issues and the way and extent to which they affect the evaluation outcomes [30]. Hence, checklist application is necessary but insufficient to analyse the methodological quality of economic evaluations and must be complemented by a narrative synthesis of methodological features. This dual approach was adopted by few previous systematic reviews in this overview (see AMSTAR 2 item 9 in Table F, Supplementary material) and hence remains a research priority for future systematic reviews.

Sole reliance on cost-per-unit ratios would generate incomplete and misleading commissioning recommendations. As noted above, single-component HAM or exercise may generate very favourable cost-per-unit ratios and yet perform poorly in terms of aggregate impact and/or coverage of priority groups relative to a multifactorial intervention. This observation contributes to an ongoing debate on whether less resource-intensive exercise should be preferred over (the widely recommended) multifactorial interventions [58, 59]. The debate is primarily centred around efficacy estimates and cost-per-unit ratios, but the final verdict cannot and should not be reached without considering the aggregate impact [60, 61] and decisional priorities beyond cost-effectiveness [62]. Consideration of aggregate outcomes is also important for informing targeting strategies (under budget/capacity constraints) and assessing the returns on intervention scale-up [26]. Systematic reviews should therefore endeavour to extract a wide range of economic evaluation outcomes, though the feasible range would largely depend on the methodological and reporting practices of underlying evaluations.

### Strengths and limitations of this systematic overview

This systematic overview is the first of its kind in the falls prevention economic evaluation context. It covered 12 academic databases and grey literature between 2003 and 2020 and followed the Cochrane guideline [20]. It offered commissioning recommendations based on general population, lifetime models after considering their methodological caveats and outcomes beyond cost-per-unit ratios. It also critically appraised the methodological quality of previous systematic reviews, and this would help improve the quality of future systematic reviews’ data extraction, quality assessment and formulation of commissioning recommendations. This would in turn aid the conceptualisation and implementation of future falls prevention economic evaluations, particularly those employing decision models.

The overview nevertheless has limitations, including non-coverage of the period before 2003, non-inclusion of systematic reviews of falls prevention RCTs that contained a minority of studies that were economic evaluations (10–12), and non-inclusion of reviews that targeted specific patient groups such as those with neurological disorders [63]. The systematic reviews of falls prevention RCTs could have contained SVEs not captured by the seven systematic reviews included in this overview. However, their methods for data extraction and synthesis and methodological appraisal would have differed substantially from the reviews that mainly targeted and included economic evaluations. Their inclusion would thus have over-extended the boundary of the review methods appraisal by this overview. The commissioning recommendations were made under certain assumptions on decision-maker preference – i.e., prioritization of general population, lifetime modelling evidence and cost-effectiveness threshold of £30,000 per QALY gained – and neglected evidence from SVEs and short-horizon models. They were also made without a comprehensive methodological appraisal of the underlying evaluations (an appropriate task for a de novo systematic review), although the key methodological caveats that may affect their outcomes were listed for each model in Table 5.

## Conclusion

The systematic overview found significant limitations in the methodological quality of existing systematic reviews of falls prevention economic evaluations which could misinform commissioning decisions and hinder the design of future evaluations. Systematic reviews should: be as comprehensive as possible in the extraction and narrative synthesis of evaluation features associated with falls epidemiology, falls prevention intervention and decision modelling; they should also base the commissioning recommendations on the full range of reported outcomes and equity objectives to avoid incomplete information being provided to decision-makers.

## Supplementary Information

 **Additional file1:** **TableA1.** Medline search strategyfor systematic overview and systematic review. **Table A2.** Embase search strategy for systematic overview ofsystematic reviews of falls prevention economic evaluation. **Table A3.** PubMed search strategy for systematic overview andsystematic review. **Table A4.** Cochrane Library (CSDR and CENTRAL) searchstrategy for systematic overview and systematic review. **Table A5.** EconLit search strategy for systematic overviewand systematic review. **Table A6.** CINAHL search strategy for systematic overview andsystematic review. **Table A7.** PsycInfo search strategy for systematic overviewand systematic review. **Table A8.** ASSIA search strategy for systematic overview andsystematic review. **Table B.** Studies excluded from systematic overview at fulltext screening and exclusion reason. **Table C.** Primary economic evaluations of community-basedfalls prevention interventions included in previous systematic reviews. **Table D.** Items contained in checklists used for qualityassessment of economic evaluations included in systematic reviews. **Table E.** Results of quality assessment by previoussystematic reviews of community-based falls prevention economic evaluations. **Table F.** AMSTAR 2 checklist for reporting andmethodological quality of systematic reviews (56).

## Data Availability

The datasets used and analysed during the current study are available from the corresponding author on reasonable request.

## Abbreviations

AMSTAR: A MeaSurement Tool to Assess systematic Reviews
CEA: Cost-effectiveness analysis
CG: Clinical guideline
CHEERS: Consolidated Health Economic Evaluation Reporting Standards
CUA: Cost-utility analysis
HAM: Home assessment and modification
HTA: Health technology assessment
ICER: Incremental cost-effectiveness ratio
MA fall: Fall requiring medical attention
PRISMA: Preferred Reporting Items for Systematic Reviews and Meta-Analyses
QALY: Quality adjusted life year
NICE: National Institute for Health and Clinical Excellence
PHE: Public Health England
RCN: Royal College of Nursing
RCT: Randomised controlled trial
SVE: Single-vehicle evaluation
