# Supplementary material for: Economic evaluation of community-based falls prevention interventions for older populations: a systematic methodological overview of systematic reviews
Source: BMC Health Serv Res. 2022 Mar 26;22:401. doi: 10.1186/s12913-022-07764-2 (PMC8962024; doi:10.1186/s12913-022-07764-2)
Supplement: Supplementary file 1 — Additional file1: TableA1. Medline search strategyfor systematic overview and systematic review. Table A2. Embase search strategy for systematic overview ofsystematic reviews of falls prevention economic evaluation. Table A3. PubMed search strategy for systematic overview andsystematic review. Table A4. Cochrane Library (CSDR and CENTRAL) searchstrategy for systematic overview and systematic review. Table A5. EconLit search strategy for systematic overviewand systematic review. Table A6. CINAHL search strategy for systematic overview andsystematic review. Table A7. PsycInfo search strategy for systematic overviewand systematic review. Table A8. ASSIA search strategy for systematic overview andsystematic review. Table B. Studies excluded from systematic overview at fulltext screening and exclusion reason. Table C. Primary economic evaluations of community-basedfalls prevention interventions included in previous systematic reviews. Table D. Items contained in checklists used for qualityassessment of economic evaluations included in systematic reviews. Table E. Results of quality assessment by previoussystematic reviews of community-based falls prevention economic evaluations. Table F. AMSTAR 2 checklist for reporting andmethodological quality of systematic reviews (56). [file 12913_2022_7764_MOESM1_ESM.docx]

# PRISMA 2020 Checklist

| **Section and Topic** | **Item #** | **Checklist item** | **Location where item is reported** |
| --- | --- | --- | --- |
| **TITLE** | | |  |
| Title | 1 | Identify the report as a systematic review [overview]. | Title |
| **ABSTRACT** | | |  |
| Abstract | 2 | See the PRISMA 2020 for Abstracts checklist. | Abstract checklist items met. |
| **INTRODUCTION** | | |  |
| Rationale | 3 | Describe the rationale for the review in the context of existing knowledge. | ‘Background’ |
| Objectives | 4 | Provide an explicit statement of the objective(s) or question(s) the review addresses. | ‘Aim and objectives’ |
| **METHODS** | | |  |
| Eligibility criteria | 5 | Specify the inclusion and exclusion criteria for the review and how studies were grouped for the syntheses. | ‘Search strategy and selection criteria’ (SSSC); ‘Data extraction and synthesis (DES) |
| Information sources | 6 | Specify all databases, registers, websites, organisations, reference lists and other sources searched or consulted to identify studies. Specify the date when each source was last searched or consulted. | Supplementary material (SM), ‘Search strategies’ |
| Search strategy | 7 | Present the full search strategies for all databases, registers and websites, including any filters and limits used. | Supplementary material (SM), ‘Search strategies’ |
| Selection process | 8 | Specify the methods used to decide whether a study met the inclusion criteria of the review, including how many reviewers screened each record and each report retrieved, whether they worked independently, and if applicable, details of automation tools used in the process. | SSSC |
| Data collection process | 9 | Specify the methods used to collect data from reports, including how many reviewers collected data from each report, whether they worked independently, any processes for obtaining or confirming data from study investigators, and if applicable, details of automation tools used in the process. | DES |
| Data items | 10a | List and define all outcomes for which data were sought. Specify whether all results that were compatible with each outcome domain in each study were sought (e.g. for all measures, time points, analyses), and if not, the methods used to decide which results to collect. | DES |
|  | 10b | List and define all other variables for which data were sought (e.g. participant and intervention characteristics, funding sources). Describe any assumptions made about any missing or unclear information. | DES |
| Study risk of bias assessment | 11 | Specify the methods used to assess risk of bias in the included studies, including details of the tool(s) used, how many reviewers assessed each study and whether they worked independently, and if applicable, details of automation tools used in the process. | ‘Critical appraisal of previous systematic review methodology’ in Methods (CA-Methods) |
| Effect measures | 12 | Specify for each outcome the effect measure(s) (e.g. risk ratio, mean difference) used in the synthesis or presentation of results. | ‘Commissioning recommendations by this systematic overview’ in Methods (CR-Methods) |
| Synthesis methods | 13a | Describe the processes used to decide which studies were eligible for each synthesis (e.g. tabulating the study intervention characteristics and comparing against the planned groups for each synthesis (item #5)). | (CR-Methods) |
|  | 13b | Describe any methods required to prepare the data for presentation or synthesis, such as handling of missing summary statistics, or data conversions. | (CR-Methods) |
|  | 13c | Describe any methods used to tabulate or visually display results of individual studies and syntheses. | CA-Methods |
|  | 13d | Describe any methods used to synthesize results and provide a rationale for the choice(s). If meta-analysis was performed, describe the model(s), method(s) to identify the presence and extent of statistical heterogeneity, and software package(s) used. | CA-Methods; CR-Methods |
|  | 13e | Describe any methods used to explore possible causes of heterogeneity among study results (e.g. subgroup analysis, meta-regression). | No analysis of heterogeneity |
|  | 13f | Describe any sensitivity analyses conducted to assess robustness of the synthesized results. | No sensitivity analysis |
| Reporting bias assessment | 14 | Describe any methods used to assess risk of bias due to missing results in a synthesis (arising from reporting biases). | CA-Methods |
| Certainty assessment | 15 | Describe any methods used to assess certainty (or confidence) in the body of evidence for an outcome. | CA-Methods |
| **RESULTS** | | |  |
| Study selection | 16a | Describe the results of the search and selection process, from the number of records identified in the search to the number of studies included in the review, ideally using a flow diagram. | Figure 1 |
|  | 16b | Cite studies that might appear to meet the inclusion criteria, but which were excluded, and explain why they were excluded. | SM, Table B |
| Study characteristics | 17 | Cite each included study and present its characteristics. | Table 2 |
| Risk of bias in studies | 18 | Present assessments of risk of bias for each included study. | ‘Critical appraisal of previous systematic review methodology’ in Results (CA-Results); SM, Table F |
| Results of individual studies | 19 | For all outcomes, present, for each study: (a) summary statistics for each group (where appropriate) and (b) an effect estimate and its precision (e.g. confidence/credible interval), ideally using structured tables or plots. | Not applicable |
| Results of syntheses | 20a | For each synthesis, briefly summarise the characteristics and risk of bias among contributing studies. | CA-Results; SM, Table F |
|  | 20b | Present results of all statistical syntheses conducted. If meta-analysis was done, present for each the summary estimate and its precision (e.g. confidence/credible interval) and measures of statistical heterogeneity. If comparing groups, describe the direction of the effect. | No statistical synthesis |
|  | 20c | Present results of all investigations of possible causes of heterogeneity among study results. | No analysis of heterogeneity |
|  | 20d | Present results of all sensitivity analyses conducted to assess the robustness of the synthesized results. | No sensitivity analysis |
| Reporting biases | 21 | Present assessments of risk of bias due to missing results (arising from reporting biases) for each synthesis assessed. | No risk of bias analysis for synthesis |
| Certainty of evidence | 22 | Present assessments of certainty (or confidence) in the body of evidence for each outcome assessed. | CA-Results; Table 3 |
| **DISCUSSION** | | |  |
| Discussion | 23a | Provide a general interpretation of the results in the context of other evidence. | ‘Discussion’ |
|  | 23b | Discuss any limitations of the evidence included in the review. | ‘Strengths and limitations of this systematic overview’ (SL) |
|  | 23c | Discuss any limitations of the review processes used. | SL |
|  | 23d | Discuss implications of the results for practice, policy, and future research. | ‘Conclusion’ |
| **OTHER INFORMATION** | | |  |
| Registration and protocol | 24a | Provide registration information for the review, including register name and registration number, or state that the review was not registered. | ‘Methods’ |
|  | 24b | Indicate where the review protocol can be accessed, or state that a protocol was not prepared. | ‘Methods’ |
|  | 24c | Describe and explain any amendments to information provided at registration or in the protocol. | No amendment made |
| Support | 25 | Describe sources of financial or non-financial support for the review, and the role of the funders or sponsors in the review. | ‘Declarations’ |
| Competing interests | 26 | Declare any competing interests of review authors. | ‘Declarations’ |
| Availability of data, code and other materials | 27 | Report which of the following are publicly available and where they can be found: template data collection forms; data extracted from included studies; data used for all analyses; analytic code; any other materials used in the review. | ‘Declarations’ |

# Search strategies

Tables A1 to A8 show the search strategies for Medline, Embase, PubMed, Cochrane Library (CDSR and CENTRAL), EconLit, CINAHL, PsycInfo and ASSIA. The search strategies for CRD, CEA Registry, PEDro and grey literature websites are shown in text.

| **Table A1** Medline search strategy for systematic overview and systematic review. | | | |
| --- | --- | --- | --- |
| Medline (Epub Ahead of Print, In-Process & Other Non-Indexed Citations, Ovid MEDLINE(R) Daily and Ovid MEDLINE(R)) – run on 7^th^ January 2021 | | | |
| **Theme** | **Type** | **Ref** | **Search Term** |
| Falls | Free text^1^ | 1 | (fall or falls or falling or faller* or fallen or fell or slip* or trip* or stumbl*).ti,ab. |
|  | MeSH | 2 | exp Accidental falls/ |
|  |  | 3 | 1 or 2 |
| Older and frailty | Free text^1^ | 4 | (old or older or senior* or elder* or aged or geriatric* or frail* or pensioner).ti,ab. |
|  | MeSH | 5 | exp Aged/ or Frailty/ |
|  |  | 6 | 4 or 5 |
| Economic evaluation | Free text^1^ | 7 | (economic or decision or Markov or cost-effectiveness or cost-utility or cost-benefit or cost or budget or expenditure or pric* or ROI).ti,ab. |
|  | MeSH | 8 | exp Models, economic/ or exp Economics/ or exp Economics, medical/ or exp Economics, nursing/ or exp Economics, pharmaceutical/ or exp Decision trees/ or exp Cost-benefit analysis/ or exp Costs and cost analysis/ or exp Budgets/ |
|  |  | 9 | 7 or 8 |
|  |  | 10 | 3 AND 6 AND 9 |
| Exclusions |  | 11 | Limit to Humans |
|  |  | 12 | (news or comment or editorial or letter or case reports).pt. or case report.ti. |
|  |  | 13 | 11 NOT 12 |
|  |  | 14 | Limit to English |
|  |  | 15 | Limit to 1^st^ January 2003 – 31^st^ December 2020 |
| **Abbreviation:** MeSH: medical subject heading; Ref: reference  ^1^ Covering title and abstract. | | | |

| **Table A2** Embase search strategy for systematic overview of systematic reviews of falls prevention economic evaluation. | | | |
| --- | --- | --- | --- |
| Embase (source: OvidSP) – run on 7^th^ January 2021 | | | |
| **Theme** | **Type** | **Ref** | **Search Term** |
| Falls | Free text^1^ | 1 | (fall or falls or falling or faller* or fallen or fell or slip* or trip* or stumbl*).ti,ab,kw. |
|  | MeSH | 2 | exp falling/ OR exp fall risk/ |
|  |  | 3 | 1 or 2 |
| Older and frailty | Free text^1^ | 4 | (old or older or senior* or elder* or aged or geriatric* or frail* or pensioner).ti,ab. |
|  | MeSH | 5 | exp Aged/ or Frailty/ |
|  |  | 6 | 4 or 5 |
| Economic evaluation | Free text^1^ | 7 | (economic OR evaluation OR budget OR expenditure OR cost* OR ROI).ti,ab,kw. |
|  | MeSH | 8 | exp Health economics/ OR exp Economic model/ OR exp Economic evaluation/ OR exp Health care cost/ OR Pharmacoeconomics/ OR Cost effectiveness analysis/ OR Cost utility analysis/ OR Cost benefit analysis/ OR Cost minimization analysis/ OR Cost of illness/ |
|  |  | 9 | 7 or 8 |
|  |  | 10 | 3 AND 6 AND 9 |
| Exclusions |  | 11 | Limit to Humans |
|  |  | 12 | (news or comment or editorial or letter or case reports).pt. or case report.ti. |
|  |  | 13 | 11 NOT 12 |
|  |  | 14 | Limit to English |
|  |  | 15 | Exclude Medline journals |
|  |  | 16 | Limit to 1^st^ January 2003 – 31^st^ December 2020 |
| **Abbreviation:** MeSH: medical subject heading; Ref: reference  ^1^ Covering title and abstract. | | | |

| **Table A3** PubMed search strategy for systematic overview and systematic review. | | | |
| --- | --- | --- | --- |
| PubMed – run on 7^th^ January 2021 | | | |
| **Theme** | **Type** | **Ref** | **Search Term** |
| Falls | Free text^1^ | 1 | [tiab] fall or falls or falling or fallen or fell or slip or trip or stumbl* |
|  | MeSH | 2 | Accidental falls |
|  |  | 3 | 1 or 2 |
| Older and frailty | Free text^1^ | 4 | [tiab] old or older or senior or elder or aged or geriatric or frail or pensioner |
|  | MeSH | 5 | Aged or Frailty |
|  |  | 6 | 4 or 5 |
| Economic evaluation | Free text^1^ | 7 | [tiab] economic or budget or expenditure or evaluation or cost or markov or model or ROI |
|  | MeSH | 8 | Model, economic or Economics, medical or Economics, nursing or Economics, pharmaceutical or Costs and cost analysis or Costs and benefits or Budget or Markov chain or Decision analysis |
|  |  | 9 | 7 or 8 |
|  |  | 10 | 3 AND 6 AND 9 |
| Exclusions |  | 11 | Limit to Humans |
|  |  | 12 | (news or comment or editorial or letter or case reports).pt. or case report.ti. |
|  |  | 13 | 11 NOT 12 |
|  |  | 14 | Limit to English |
|  |  | 15 | Remove [Child: Birth-18 years], [Infant: 1-23 months] |
|  |  | 16 | Limit to 1^st^ January 2003 – 31^st^ December 2020 |
| **Abbreviation:** MeSH: medical subject heading; Ref: reference; tiab: titles and abstract  ^1^ Covering title and abstract. | | | |

| **Table A4** Cochrane Library (CSDR and CENTRAL) search strategy for systematic overview and systematic review. | | | |
| --- | --- | --- | --- |
| Cochrane Library (Cochrane Database of Systematic Reviews and CENTRAL trials registry) – run on 7^th^ January 2021 | | | |
| **Theme** | **Type** | **Ref** | **Search Term** |
| Falls | Free text^1^ | 1 | (fall or falls or falling or fallen or fell or slip or trip or stumbl*).ti,ab,kw. |
|  | MeSH | 2 | exp Accidental falls/ |
|  |  | 3 | 1 or 2 |
| Older and frailty | Free text^1^ | 4 | (old or older or senior or elder or aged or geriatric or frail or pensioner).ti,ab,kw. |
|  | MeSH | 5 | exp Aged/ or exp Aging/ or exp Frailty/ or exp Frail elderly/ |
|  |  | 6 | 4 or 5 |
| Economic evaluation | Free text^1^ | 7 | (economic OR evaluation OR budget OR expenditure OR cost* OR ROI).ti,ab,kw. |
|  | MeSH | 8 | exp Economics/ or exp Economics, nursing/ or exp Economics, pharmaceutical/ or exp Economics, medical/ or exp Models, Economic/ or exp Costs and cost analysis/ or exp Cost-benefit analysis/ or exp Cost of illness/ or exp Budgets/ or exp Health expenditures/ |
|  |  | 9 | 7 or 8 |
|  |  | 10 | 3 AND 6 AND 9 |
| Exclusions |  | 11 | Limit to 1^st^ January 2003 – 31^st^ December 2020 |
| **Abbreviation:** MeSH: medical subject heading; Ref: reference  ^1^ Covering title and abstract. | | | |

| **Table A5** EconLit search strategy for systematic overview and systematic review. | | | |
| --- | --- | --- | --- |
| EconLit (source: OvidSP) – run on 7^th^ January 2021 | | | |
| **Theme** | **Type** | **Ref** | **Search Term** |
| Falls | Free text^1^ | 1 | (fall or falls or falling or fallen or fell or slip or trip or stumbl*).ti,ab,kw. |
| Older and frailty | Free text^1^ | 2 | (old or older or senior or elder or aged or geriatric or frail or pensioner).ti,ab,kw. |
| Economic evaluation | Free text^1^ | 3 | (economic OR evaluation OR budget OR expenditure OR cost* OR ROI).ti,ab,kw. |
|  |  | 4 | 1 AND 2 AND 3 |
| Exclusions |  | 5 | Limit to English |
|  |  | 6 | Limit to 1^st^ January 2003 – 31^st^ December 2020 |
| **Abbreviation:** MeSH: medical subject heading; Ref: reference  ^1^ Covering title and abstract. | | | |

| **Table A6** CINAHL search strategy for systematic overview and systematic review. | | | |
| --- | --- | --- | --- |
| CINAHL (source: EBSCO) – run on 7^th^ January 2021 | | | |
| **Theme** | **Type** | **Ref** | **Search Term** |
| Falls | Free text^1^ | 1 | TI(fall or falls or falling or fallen or fell or slip or trip or stumbl*) |
|  | Free text | 2 | AB(fall or falls or falling or fallen or fell or slip or trip or stumbl*) |
|  | MeSH | 3 | MH(Accidental falls) |
|  |  | 4 | 1 or 2 or 3 |
| Older and frailty | Free text^1^ | 5 | TI(old or older or senior or elder or aged or geriatric or frail or pensioner) |
|  | Free text | 6 | AB(old or older or senior or elder or aged or geriatric or frail or pensioner) |
|  | MeSH | 7 | MH(Aged+) |
|  |  | 8 | 5 or 6 or 7 |
| Economic evaluation | Free text^1^ | 9 | TI(economic or evaluation or budget or expenditure or cost* or ROI) |
|  | Free text | 10 | AB(economic or evaluation or budget or expenditure or cost* or ROI) |
|  | MeSH | 11 | MH(Economics or Economic aspects of illness or Economics, pharmaceutical or Accidental falls economics) |
|  |  | 12 | 9 or 10 or 11 |
|  |  | 13 | 4 AND 8 AND 12 |
| Exclusions |  | 14 | Limit to Humans |
|  |  | 15 | PT(news or comment or editorial or letter or case reports) |
|  |  | 16 | 14 NOT 15 |
|  |  | 17 | Limit to English |
|  |  | 18 | Limit to Academic Journals (remove Dissertations, Magazines and CEUs) |
|  |  | 19 | Limit to 1^st^ January 2003 – 31^st^ December 2020 |
| **Abbreviation:** AB: abstract; MeSH: medical subject heading; Ref: reference; TI: title.  ^1^ Covering title and abstract. | | | |

| **Table A7** PsycInfo search strategy for systematic overview and systematic review. | | | |
| --- | --- | --- | --- |
| PsycInfo (source: OvidSP) – run on 7^th^ January 2021 | | | |
| **Theme** | **Type** | **Ref** | **Search Term** |
| Falls | Free text^1^ | 1 | (fall or falls or falling or fallen or fell or slip or trip or stumbl*).ti,ab. |
|  | MeSH | 2 | Falls/ |
|  |  | 3 | 1 or 2 |
| Older and frailty | Free text^1^ | 4 | (old or older or senior or elder or aged or geriatric or frail or pensioner).ti,ab. |
|  | MeSH | 5 | exp Aging/ or Geriatrics/ or Gerontology/ |
|  |  | 6 | 4 or 5 |
| Economic evaluation | Free text^1^ | 7 | (economic OR evaluation OR budget OR expenditure OR cost* OR ROI).ti,ab. |
|  | MeSH | 8 | exp Economics/ or exp “Costs and cost analysis”/ or “Resource allocation”/ or exp “Decision making”/ |
|  |  | 9 | 7 or 8 |
|  |  | 10 | 3 AND 6 AND 9 |
| Exclusions |  | 11 | Limit to Humans |
|  |  | 12 | (news or comment or editorial or letter or case reports).pt. or case report.ti. |
|  |  | 13 | 11 NOT 12 |
|  |  | 14 | Limit to English |
|  |  | 15 | Limit to 1^st^ January 2003 – 31^st^ December 2020 |
| **Abbreviation:** MeSH: medical subject heading; Ref: reference  ^1^ Covering title and abstract. | | | |

| **Table A8** ASSIA search strategy for systematic overview and systematic review. | | | |
| --- | --- | --- | --- |
| ASSIA (source: ProQuest) – run on 7^th^ January 2021 | | | |
| **Theme** | **Type** | **Ref** | **Search Term** |
| Falls | Free text^1^ | 1 | ti(fall or falls or falling or fallen or fell or slip or trip or stumbl*) |
|  | Free text | 2 | ab(fall or falls or falling or fallen or fell or slip or trip or stumbl*) |
|  | SH | 3 | Mainsubject.Exact(“falls” OR “accidental falls” OR “fall prone elderly people”) |
|  |  | 4 | 1 or 2 or 3 |
| Older and frailty | Free text^1^ | 5 | ti(old or older or senior or elder or aged or geriatric or frail or pensioner) |
|  | Free text | 6 | ab(old or older or senior or elder or aged or geriatric or frail or pensioner) |
|  | SH | 7 | Mainsubject.Exact(“aged, 80 & over” or “aged” or “frailty” or “frail elderly” or “frail elderly people” or “frail”) |
|  |  | 8 | 5 or 6 or 7 |
| Economic evaluation | Free text^1^ | 9 | ti(economic or evaluation or budget or expenditure or cost* or ROI) |
|  | Free text | 10 | ab(economic or evaluation or budget or expenditure or cost* or ROI) |
|  | SH | 11 | Mainsubject.Exact(“economic costs” or “economic aspects” or “economic analysis” or “economic impact” or “economic” or “budgets” or “benefit cost analysis” or “costs” or “cost-benefit analysis” or “cost effectiveness” or “costing” or “cost utility analysis” or “cost minimization analysis” or “cost benefit analysis” or “costs & cost analysis” or “cost analysis” or “cost of illness”) |
|  |  | 12 | 9 or 10 or 11 |
|  |  | 13 | 4 AND 8 AND 12 |
| Exclusions |  | 14 | Exclude commentary, news and editorial |
|  |  | 15 | Limit to English |
|  |  | 16 | Limit to 1^st^ January 2003 – 31^st^ December 2020 |
| **Abbreviation:** AB: abstract; Ref: reference; SH: subject heading; TI: title.  ^1^ Covering title and abstract. | | | |

**Other databases**

Search strategy for Centre for Reviews and Dissemination (CRD) – search run on 7^th^ January 2021

Title: “Fall” AND Limit publication year to 2003-2020

Result #: 61

Search strategy for Cost-Effectiveness Analysis (CEA) Registry – search run on 7^th^ January 2021

“Fall” as search term

Result #: 100 (only the most recent 100 hits available)

Search strategy for Physiotherapy Evidence Database (PEDro) – search run on 7^th^ January 2021

Title: “Fall” AND Limit publication year to 2003-2020

Result #: 226

**Grey literature**

The following sites were searched with term “Falls prevention”

- Age UK: <https://www.ageuk.org.uk/>
- Chartered Society of Physiotherapy: <https://www.csp.org.uk/>
- College of Occupational Therapy: <https://www.rcot.co.uk/>
- Department of Health: <https://www.gov.uk/government/organisations/department-of-health-and-social-care>
- Royal College of Nursing: <https://www.rcn.org.uk/>

# Excluded studies

| **Table B** Studies excluded from systematic overview at full text screening and exclusion reason. | | |
| --- | --- | --- |
| **First author (year)** | **Title** | **Main exclusion reason** |
| Annweiler (2010) | Fall prevention and vitamin D in the elderly: an overview of the key role of the non-bone effects | General review on falls epidemiology and prevention |
| Arnold (2008) | Exercise for fall risk reduction in community-dwelling older adults: a systematic review | SR with <50% of studies that are FP economic evaluations |
| Avenell (2014) | Vitamin D and vitamin D analogues for preventing fractures in post‐menopausal women and older men | SR with <50% of studies that are FP economic evaluations |
| Beswick (2010) | Complex interventions to improve physical function and maintain independent living in elderly people: a systematic review and meta-analysis | SR with <50% of studies that are FP economic evaluations |
| Bischoff-Ferrari (2004) | Effect of Vitamin D on Falls: A Meta-analysis | SR with <50% of studies that are FP economic evaluations |
| Bischoff-Ferrari (2009) | Fall prevention with supplemental and active forms of vitamin D: a meta-analysis of randomised controlled trials | SR with <50% of studies that are FP economic evaluations |
| Boonen (2006) | Addressing the musculoskeletal components of fracture risk with calcium and vitamin D: a review of the evidence | General review on falls epidemiology and prevention |
| Boye (2013) | The impact of falls in the elderly | General review on falls epidemiology and prevention |
| Boyle (2010) | Medication and falls: risk and optimization | General review on falls epidemiology and prevention |
| Campbell (2010) | Comprehensive approach to fall prevention on a national level: New Zealand | General review on falls epidemiology and prevention |
| Chang (2004) | Interventions for the prevention of falls in older adults: systematic review and meta-analysis of randomised clinical trials | SR with <50% of studies that are FP economic evaluations |
| Chase (2012) | Systematic review of the effect of home modification and fall prevention programs on falls and the performance of community-dwelling older adults | SR with <50% of studies that are FP economic evaluations |
| Clemson (2008) | Environmental interventions to prevent falls in community-dwelling older people: a meta-analysis of randomized trials | SR with <50% of studies that are FP economic evaluations |
| Gillespie (2012) | Interventions for preventing falls in older people living in the community | SR with <50% of studies that are FP economic evaluations |
| Goodwin (2014) | Multiple component interventions for preventing falls and fall-related injuries among older people: systematic review and meta-analysis | SR with <50% of studies that are FP economic evaluations |
| Guirguis-Blake (2018) | Interventions to prevent falls in older adults: updated evidence report and systematic review for the US Preventive Services Task Force | SR with <50% of studies that are FP economic evaluations |
| Hackney (2014) | Impact of Tai Chi Chu'an practice on balance and mobility in older adults: an integrative review of 20 years of research | General review on falls epidemiology and prevention |
| Hanley (2011) | Community-based health efforts for the prevention of falls in the elderly | General review on falls epidemiology and prevention |
| Hempel (2014) | Evidence map of Tai Chi | General review on falls epidemiology and prevention |
| Hiligsmann (2015) | A systematic review of cost-effectiveness analyses of drugs for postmenopausal osteoporosis | SR with <50% of studies that are FP economic evaluations |
| Hill (2012) | Psychotropic drug-induced falls in older people | General review on falls epidemiology and prevention |
| Hopewell (2018) | Multifactorial and multiple component interventions for preventing falls in older people living in the community | SR with <50% of studies that are FP economic evaluations |
| Huang (2012) | Medication-related falls in the elderly | General review on falls epidemiology and prevention |
| Karinkanta (2010) | Physical therapy approaches to reduce fall and fracture risk among older adults | General review on falls epidemiology and prevention |
| Karlsson (2013) | Prevention of falls in the elderly—a review | General review on falls epidemiology and prevention |
| Lord (2006) | Home environment risk factors for falls in older people and the efficacy of home modifications | General review on falls epidemiology and prevention |
| Lord (2010) | Vision and falls in older people: risk factors and intervention strategies | General review on falls epidemiology and prevention |
| Marcelli (2015) | Beneficial effects of vitamin D on falls and fractures: is cognition rather than bone or muscle behind these benefits? | General review on falls epidemiology and prevention |
| Montero-Odasso (2018) | Falls in cognitively impaired older adults: implications for risk assessment and prevention | General review on falls epidemiology and prevention |
| Nowak (2009) | Falls and frailty: lessons from complex systems | General review on falls epidemiology and prevention |
| Pega (2016) | A systematic review of health economic analyses of housing improvement interventions and insecticide-treated bednets in the home | SR with <50% of studies that are FP economic evaluations |
| Pisani (2016) | Major osteoporotic fragility fractures: Risk factor updates and societal impact | General review on falls epidemiology and prevention |
| Pynoos (2010) | Environmental assessment and modification as fall-prevention strategies for older adults | General review on falls epidemiology and prevention |
| Reed-Jones (2013) | Vision and falls: a multidisciplinary review of the contributions of visual impairment to falls among older adults | General review on falls epidemiology and prevention |
| Rose (2008) | Preventing falls among older adults: No "one size suits all" intervention strategy | General review on falls epidemiology and prevention |
| Rubenstein (2006) | Falls and their prevention in elderly people: what does the evidence show? | General review on falls epidemiology and prevention |
| Rubenstein (2006b) | Falls in older people: epidemiology, risk factors and strategies for prevention | General review on falls epidemiology and prevention |
| Shaw (2007) | Prevention of falls in older people with dementia | General review on falls epidemiology and prevention |
| Sherrington (2017) | Exercise to prevent falls in older adults: an updated systematic review and meta-analysis | SR with <50% of studies that are FP economic evaluations |
| Sleet (2008) | CDC's research portfolio in older adult fall prevention: a review of progress, 1985-2005, and future research directions | General review on falls epidemiology and prevention |
| Soriano (2007) | Falls in the community-dwelling older adult: a review for primary-care providers | General review on falls epidemiology and prevention |
| Tinetti (2003) | Preventing falls in elderly persons | General review on falls epidemiology and prevention |
| Tinetti (2006) | Fall-risk evaluation and management: challenges in adopting geriatric care practices | General review on falls epidemiology and prevention |
| Tinetti (2010) | The patient who falls: “It's always a trade-off” | General review on falls epidemiology and prevention |
| Tofthagen (2012) | Strength and balance training for adults with peripheral neuropathy and high risk of fall: current evidence and implications for future research | General review on falls epidemiology and prevention |
| Tricco (2017) | Comparisons of interventions for preventing falls in older adults: a systematic review and meta-analysis | SR with <50% of studies that are FP economic evaluations |
| Ungar (2013) | Fall prevention in the elderly | General review on falls epidemiology and prevention |
| Vieira (2016) | Prevention of falls in older people living in the community | General review on falls epidemiology and prevention |
| **Abbreviation:** FP: falls prevention; SR: systematic review. | | |

# Primary economic evaluations identified by previous systematic reviews

| **Table C** Primary economic evaluations of community-based falls prevention interventions included in previous systematic reviews. | | | | | | | | |
| --- | --- | --- | --- | --- | --- | --- | --- | --- |
| **#** | **Economic evaluation** | **Systematic review^1^** | **Target population** | **Type of analysis** | **Perspective** | **Time horizon (Model type)** | **Intervention** | **Comparator** |
| ***Single-vehicle evaluations (SVEs) (e.g., alongside randomised controlled trial)*** | | | | | | | | |
| 1 | Campbell (2005) (1) | Davis; PHE; Olij | New Zealand CD adults aged 75+ with severe visual impairment | CEA | Societal | 1 year | Home assessment and modification (HAM)^2^ | Usual care |
| 2 | Cockayne (2017) (2) | Olij | UK and Ireland CD adults aged 65+ with fall in past year, hospitalised fall in past two years or FoF | CUA | PS; Societal | 1 year | Multiple-component podiatry | Usual care |
| 3 | Davis (2011) (3) | DJ; Olij; Winser | Canadian CD adults aged 65 to 75 | CEA; CUA | PS | 1 year | Once-weekly resistance training; twice-weekly resistance training | Twice-weekly resistance training with tone classes |
| 4 | Davis (2011b) (4) | Winser | Canadian CD adults aged 65 to 75: follow-up study to Davis (2011) | CUA | PS | 1 year | (In preceding trial) Once-weekly resistance training; twice-weekly resistance training | Twice-weekly balance and tone classes |
| 5 | Farag (2015) (5) | Olij | Australian CD adults aged 60+ recently discharged from hospital | CEA; CUA | PS | 1 year | Home exercise | Usual care |
| 6 | Farag (2016) (6) | PHE | Australian CD Parkinson’s disease patients, aged 40+ | CEA; CUA | PS | 6 months | Minimally supervised exercise | Usual care |
| 7 | Fletcher (2012) (7) | PHE | UK Parkinson’s disease patients (mean age 71) with 2+ falls in past 12 months | CUA | PS | 20 weeks | Group and home exercise | Usual care |
| 8 | Hendriks (2008) (8) | DJ; PHE; Olij; Winser | Dutch CD adults aged 65+ who experienced a fall requiring A&E/GP attention | CEA; CUA | Societal | 1 year | Multifactorial risk assessment (MRA) | Usual care |
| 9 | Irvine (2010) (9) | DJ; PHE; Olij; Winser | UK CD adults aged 70+ screened as high falls risk by GPs | CEA | PS | 1 year | Multifactorial int. | Usual care |
| 10 | Isaranuwatchai (2017) (10) | Huter; Winser | Canadian CD adults aged 75+ screened as high falls risk | CEA | Societal | 6 months | Multifactorial int. | Usual care |
| 11 | Jenkyn (2012) (11) | DJ; PHE; Olij | Canadian CD older veterans screened as high falls risk by postal questionnaire | CEA | Societal | 1 year | MRA | Usual care |
| 12 | Kenkre (2002) (12) | DJ | UK CD adults aged 65+ | ROI | PS | 1 year | Falls prevention education | Usual care |
| 13 | Li (2015) (13) | PHE | US CD mild-to-moderate Parkinson’s disease patients aged 40-85 | CEA; CUA | Societal | 9 months | Tai Chi | Resistance exercise; Stretching |
| 14 | Patil (2016) (14) | PHE; Olij | Finnish CD women aged 70-80 who have fallen at least once in past year and low physical activity | CEA | Societal | 2 years | Exercise and Vit. D; Exercise alone; Vit. D alone | Usual care |
| 15 | Peeters (2011) (15) | PHE; Olij | Dutch CD and RC adults aged 65+ screened as high falls risk by questionnaire | CEA; CUA | Societal | 1 year | Multifactorial int. | Usual care |
| 16 | Polinder (2016) (16) | PHE | Dutch CD adults aged 65+ who visited A&E after a fall and use fall-risk-increasing medications | CUA | Societal | 1 year | Med. modification | Usual care |
| 17 | Rizzo (1996) (17) | RCN; Davis; Olij | US adults aged 70+ with one or more of 8 falls risk factors | CEA; ROI | US healthcare^5^ | 1 year | Multifactorial int. | Usual care |
| 18 | Robertson (2001a) (18) | RCN; Davis; DJ; Olij | New Zealand CD adults aged 75+ | CEA | PS^3^ | 1 year | Home exercise (delivered by nurse home visits) | Usual care |
| 19 | Robertson (2001b) (19) | RCN; Davis; DJ; Olij | New Zealand CD adults aged 80+ | CEA | PS | 1 year | Centre-based exercise | Usual care |
| 20 | Robertson (2001c) (20) | Davis; DJ; Olij | New Zealand CD women aged 80+ | CEA | PS^3^ | 2 years | Home exercise (individually tailored) | Usual care |
| 21 | Sach (2012) (21) | PHE | UK CD and RC adults aged 60+ who have fallen and called ambulance but do not need A&E/inpatient stay | CEA; CUA | PS; Societal | 1 year | Referral to community-based multifactorial intervention following NICE guideline by ambulance paramedics | Usual care |
| 22 | Salkeld (2000) (22) | RCN; Davis; DJ; Olij | Australian CD adults aged 65+ recruited mostly from inpatient setting | CEA | Societal | 1 year | HAM delivered by occupational therapist | Usual care |
| 23 | Timonen (2008) (23) | Winser | Finnish CD women aged 75+ with mobility and balance issues admitted for acute illness to primary care | ROI | PS | 1 year | Group-based exercise program | Usual care |
| ***Decision models*** | | | | | | | | |
| 1 | Albert (2016) (24) | PHE | US CD adults aged 50+ (mean age 75.5) | CUA | PS | 1 year (DT) | Multifactorial int. | Usual care |
| 2 | Beard (2006) (25) | Davis; PHE | Australian CD adults aged 60+ | ROI; CBA | PS; Societal | 5 years (Binary^4^) | Multiple-component (intersectoral) int. | Usual care |
| 3 | Carande-Kulis (2015) (26) | DJ; PHE | US CD adults aged 65+ | ROI | US healthcare^5^ | 1 year (Binary) | Exercise (2 forms); Multiple-component int. (Stepping On) | Usual care |
| 4 | Church (2011) (27) | DJ; PHE; Olij | Australian CD and RC adults aged 65+ | CEA; CUA | PS | 10 years (Markov cohort) | Exercise (3 forms); Stepping On; Multifactorial int.; MRA; Exp. cataract surgery; Med. modification; Cardiac pacing | Usual care |
| 5 | Church (2012) (28) | DJ; PHE; Olij | US CD adults aged 65+ | CEA; CUA | PS | Lifetime (Markov cohort) | Exercise (4 forms); Multiple-component int.; Multifactorial int. (2 forms); MRA; HAM; Exp. cataract surgery; Med. modification; Cardiac pacing | Usual care; Comparison between int. |
| 6 | Day (2009) (29) | DJ | Australian CD adults aged 50+ (age and characteristics differ by intervention type)^6^ | CEA | PS | 1 year (DT) | Exercise (2 forms); HAM; Multifactorial int.; Med. modification; Cardiac pacing | Usual care |
| 7 | Farag (2015b) (30) | DJ; PHE; Olij | Australian CD adults aged 65+ without falls history | CUA | PS | Lifetime (Markov cohort) | Non-specific intervention | Usual care |
| 8 | Frick (2010) (31) | DJ; PHE; Olij | US CD adults aged 65+ | CUA | PS | 1 year (Binary) | Exercise (2 forms); HAM; Multifactorial int. (2 forms); Med. modification; Vit. D | Comparison between int. |
| 9 | Hektoen (2009) (32) | DJ; PHE | Norwegian CD women aged 80+ | CEA | Societal | 1 year (Binary) | Exercise | Usual care |
| 10 | Johansson (2008) (33) | DJ | Swedish CD adults aged 65+ | CUA | Societal | Lifetime (Markov cohort) | Multiple-component (intersectoral) int. | Usual care |
| 11 | Lee (2013) (34) | Olij | US CD adults aged 65-80 without falls history | CBA | PS | 3 years (DT + Markov cohort) | Targeted Vit. D; Universal Vit. D | Usual care; Comparison between int. |
| 12 | Ling (2008) (35) | DJ | US CD adults aged 65+ with falls history or other risk factors | ROI | US healthcare^5^ | 1 year (Binary) | HAM | Usual care |
| 13 | McLean (2015) (36) | PHE; Olij; Huter; Winser | Australian CD adults aged 70+ | CEA; CUA | PS | 18 months (DT) | Exercise | Usual care |
| 14 | Mori (2017) (37) | Olij | US CD women aged 65+ without previous osteoporotic fracture | CUA | Societal | Lifetime (DT + Markov patient) | Exercise (alone or with bisphosphonate) | Comparison between int. |
| 15 | OMAS (2008) (38) | DJ | Canadian CD adults aged 65+ | CEA; ROI | PS | Lifetime (Markov cohort) | Exercise; HAM; Vit. D and calcium; Med. modification; gait-stabilizing device | Usual care |
| 16 | Pega (2016) (39) | PHE; Olij | New Zealand CD adults aged 65+ | CUA | PS | Lifetime (Markov cohort) | HAM | Usual care |
| 17 | Poole (2015) (40) | Olij | UK CD adults aged 60+ | CUA; ROI | PS | 5 years (Markov cohort) | Vit. D | Usual care |
| 18 | Sach (2007) (41)^7^ | Davis; Olij | UK women aged 70+ with bilateral cataracts | CEA; CUA | PS; Societal | Lifetime extrapolation (Binary) | Exp. cataract surgery (first eye) | Routine cataract surgery |
| 19 | Smith (1998) (42) | RCN; Davis; Olij | Australian CD adults aged 75+ | CEA | PS | 10 years (DT + Markov cohort) | HAM | Usual care |
| 20 | van der Velde (2008) (43) | PHE | Dutch CD geriatric outpatient population with falls history (mean age 78) | CEA | PS | 1 year (Binary) | Med. modification | Usual care |
| 21 | Wu (2010) (44) | PHE | US CD Medicare beneficiaries aged 65+ with falls history | CEA; ROI | PS; Societal | 1 year (Binary) | Multifactorial int. | Usual care |
| **Abbreviations:** CB: community-based; CBA: cost-benefit analysis; CEA: cost-effectiveness analysis; CUA: cost-utility analysis; DJ: Dubas-Jakobczyk; DT: decision tree; FoF: fear of falling; HAM: home assessment and modification; Int.: intervention; Med.: medication; MRA: multifactorial risk assessment without tailored treatments; NICE: National Institute for Health and Care Excellence; OMAS: Ontario Medical Advisory Secretariat; PHE: Public Health England; PS: public sector; RC: residential care; RCN: Royal College of Nursing; RCT: randomized controlled trial; ROI: return on investment; Vit. D: vitamin D supplementation  ^1^ References for systematic reviews: RCN (45); Davis (46); DJ (47); PHE (48); Olij (49); Huter (50); Winser (51)  ^2^ The study also included exercise and exercise and HAM, but economic evaluation was conducted only on HAM.  ^3^ The study classified itself as a societal analysis but contained no societal resource/cost items.  ^4^ Binary decision models include two scenarios, with and without intervention, and no time-based cycles or probability trees.  ^5^ These US-based studies did not specify public Medicare/Medicaid as the main payer. The payers would hence include private health insurances and patients.  ^6^ Cardiac pacing targeted population aged 50+ due to their high falls risk. Other interventions targeted populations aged 65+.  ^7^ This study was included as a decision model because it extrapolated the results of a trial over a lifetime horizon. | | | | | | | | |

# Quality assessment checklists used by previous systematic reviews

| **Table D** Items contained in checklists used for quality assessment of economic evaluations included in systematic reviews. | | | | |
| --- | --- | --- | --- | --- |
|  | **Drummond checklist (52)** | **QHES checklist (53)** | **NICE checklist (adapted) (54)** | **CHEC checklist (adapted) (55)** |
| Review | Davis review (46); DJ review (47) | Davis review (46); Winser review (51) | PHE review (48) | Olij review (49) |
| Item # | 10 | 15 (Davis); 16 (Winser) | 19 | 20 |
| Item score | (DJ review) Yes [1]; No [0]; Unclear [0]; N/A [1] | Yes; No [Points per item] | Yes; No; Partly; Unclear; N/A | Yes [1]; Suboptimal [0.5]; No [0]; N/A |
| Overall score/grade | 10  (DJ review) ‘Good’ if 9-10; ‘Moderate’ if 6-8; ‘Poor’ if 0-5 | 99 (Davis); 100 (Winser)  ‘Good’ if total score +75% | Applicability: directly; partially; not  Limitations: minor; potentially serious; very serious | 20 converted to % |
| Items | (1) Well-defined research question | (1) Study objectives clearly presented [7 points] | (1) Relevant study population for topic | (1) Study population clearly described |
|  | (2) Comprehensive description of alternatives | (2) Select perspective reasonably [4] | (2) Appropriate interventions for topic | (2) Competing alternatives clearly described |
|  | (3) Well-estimated effectiveness | (3) Best source for input estimates [8] | (3) Relevant to UK context | (3) Well-defined research question posed in answerable form |
|  | (4) All relevant costs and effects identified | (4) Subgroups prespecified at start of study [1] | (4) Perspective clearly stated | (4) Appropriate economic study design for stated objective |
|  | (5) All relevant costs and effects measured accurately | (5) Sensitivity analysis conducted to assess uncertainty & assumptions [9] | (5) All direct health and other effects included | (5) Model structural assumptions and validity properly reported |
|  | (6) All relevant costs and effects valued credibly | (6) Appropriate incremental analysis conducted [6] | (6) Appropriate discounting of future costs and outcomes | (6) Appropriate time horizon for costs and consequences |
|  | (7) Account for differential timing of costs and effects | (7) Methods for data use/abstraction clearly stated [5] | (7) Value health effects in QALYs | (7) Appropriate perspective |
|  | (8) Appropriate incremental analysis conducted | (8) Appropriate time horizon and discounting [7] | (8) Non-health costs/outcomes appropriately measured/valued | (8) All important and relevant costs for alternatives identified |
|  | (9) Allowance for uncertainty | (9) Appropriate cost measurement and unit costs described [8] | (9) Valid model structure for topic | (9) All costs measured in physical units |
|  | (10) Include all issues for evaluation users in results and discussion | (10) Primary outcome measures clearly stated with justification [6] | (10) Appropriate time horizon for costs and outcomes | (10) All costs valued appropriately |
|  |  | (11) Valid health outcome measures used [7] | (11) All relevant outcomes included | (11) All important and relevant outcomes for alternatives identified |
|  |  | (12) Model structure clearly presented [8] | (12) Baseline health outcome estimates from best available source | (12) All outcomes measured appropriately |
|  |  | (13) Main model assumptions and limitations of model justified [7] | (13) Effectiveness estimates from best available source | (13) CUA/CBA outcomes valued appropriately |
|  |  | (14) Discuss direction and magnitude of potential biases [6] | (14) All important and relevant costs included | (14) Conduct appropriate incremental analysis |
|  |  | (15) State recommendations and conclusions based on results [8] | (15) Resource use estimates from best available source | (15) Discount all future costs and outcomes appropriately |
|  |  | (16) Disclose source of funding [3] | (16) Unit cost estimates from best available source | (16) Uncertain variable values subjected to sensitivity analysis |
|  |  |  | (17) Present appropriate incremental analysis | (17) Conclusions that follow data |
|  |  |  | (18) Conduct sensitivity analysis | (18) Discuss generalizability of results to other settings/patients |
|  |  |  | (19) Report potential conflict of interest | (19) Report potential conflict of interest |
|  |  |  |  | (20) Discuss ethical and distributional issues |
| **Abbreviation:** CBA: cost-benefit analysis; CHEC: Consensus on Health Economic Criteria; CUA: cost-utility analysis; NICE: National Institute for Health and Care Excellence; QALY: quality-adjusted life-year; QHES: Quality of Health Economics Studies | | | | |

# Quality assessment checklist scores

| **Table E** Results of quality assessment by previous systematic reviews of community-based falls prevention economic evaluations | | | | | | | | | | |
| --- | --- | --- | --- | --- | --- | --- | --- | --- | --- | --- |
| **#** | **Primary study** | **Systematic review^1^** | **Quality assessment results by checklist^2^** | | | | | | | |
|  |  |  | Davis Drummond (52) | DJ/Huter Drummond (52) | Davis QHES (53)^3^ | Winser QHES (53) | PHE NICE (Yes #) (54)^4^ | PHE NICE verdict (54) | Olij CHEC (55)^6^ | Standard deviation of % scores |
|  |  |  | Max: 10 items converted % | Max: 10 items converted % | Max: 100% | Max: 100% | Max: 19 items converted % | Categories^5^ | Max: 100% |  |
| ***Single-vehicle evaluations (SVEs) (e.g., alongside randomized controlled trials)*** | | | | | | | | | | |
| 1 | Campbell (2005) (1) | Davis; PHE; Olij | 90.0 |  | 100 |  | 47.4 | Partially / Minor | 78.0 | 22.8 |
| 2 | Cockayne (2017) (2) | Olij |  |  |  |  |  |  | 63.0 |  |
| 3 | Davis (2011) (3) | DJ; Olij; Winser |  | 100 |  | 99.0 |  |  | 89.0 | 6.1 |
| 4 | Davis (2011b) (4) | Winser |  |  |  | 87.0 |  |  |  |  |
| 5 | Farag (2015) (5) | Olij |  |  |  |  |  |  | 92.0 |  |
| 6 | Farag (2016) (6) | PHE |  |  |  |  | 57.9 | Partially / Minor |  |  |
| 7 | Fletcher (2012) (7) | PHE |  |  |  |  | 57.9 | Directly / Minor |  |  |
| 8 | Hendriks (2008) (8) | DJ; PHE; Olij; Winser |  | 100 |  | 93.0 | 84.2 | Partially / Minor | 89.0 | 6.7 |
| 9 | Irvine (2010) (9) | DJ; PHE; Olij; Winser |  | 100 |  | 79.0 | 52.6 | Partially / Minor | 92.0 | 20.7 |
| 10 | Isaranuwatchai (2017) (10) | Huter; Winser |  | 70.0 |  | 91.0 |  |  |  | 14.8 |
| 11 | Jenkyn (2012) (11) | DJ; PHE; Olij |  | 90.0 |  |  | 42.1 | Partially / Minor | 79.0 | 25.1 |
| 12 | Kenkre (2002) (12) | DJ |  | 50.0 |  |  |  |  |  |  |
| 13 | Li (2015) (13) | PHE |  |  |  |  | 57.9 | Partially / Minor |  |  |
| 14 | Patil (2016) (14) | PHE; Olij |  |  |  |  | 52.6 | Partially / Minor | 97.0 | 31.4 |
| 15 | Peeters (2011) (15) | PHE; Olij |  |  |  |  | 73.7 | Directly / Minor | 92.0 | 13.0 |
| 16 | Polinder (2016) (16) | PHE |  |  |  |  | 63.2 | Directly / Minor |  |  |
| 17 | Rizzo (1996) (17) | RCN; Davis; Olij | 80.0 |  | 75.0 | 61.0 |  |  | 62.0 | 9.5 |
| 18 | Robertson (2001a) (18) | RCN; Davis; DJ; Olij | 90.0 | 80.0 | 100 | 66.0 |  |  | 91.0 | 13.0 |
| 19 | Robertson (2001b) (19) | RCN; Davis; DJ; Olij | 90.0 | 90.0 | 92.0 |  |  |  | 91.0 | 1.0 |
| 20 | Robertson (2001c) (20) | Davis; DJ; Olij | 90.0 | 80.0 | 100 | 93.0 |  |  | 94.0 | 7.3 |
| 21 | Sach (2012) (21) | PHE |  |  |  |  | 68.4 | Directly / Minor |  |  |
| 22 | Salkeld (2000) (22) | RCN; Davis; DJ; Olij | 60.0 | 80.0 | 73.0 |  |  |  | 85.0 | 10.8 |
| 23 | Timonen (2008) (23) | Winser |  |  |  | 39.0 |  |  |  |  |
|  |  | *Average:* | 83.3 | 84.0 | 90.0 | 78.7 | 59.8 |  | 85.3 | 11.5 |
| ***Decision models*** | | | | | | | | | | |
| 1 | Albert (2016) (24) | PHE |  |  |  |  | 47.4 | Partially / Potentially serious |  |  |
| 2 | Beard (2006) (25) | Davis; PHE | 70.0 |  | 59.0 |  | 52.6 | Directly / Minor |  | 8.8 |
| 3 | Carande-Kulis (2015) (26) | DJ; PHE |  | 80.0 |  |  | 42.1 | Partially / Minor |  | 26.8 |
| 4 | Church (2011) (27) | DJ; PHE; Olij |  | 70.0 |  |  | 78.9 | Partially / Minor | 83.0 | 6.7 |
| 5 | Church (2012) (28) | DJ; PHE; Olij |  | 50.0 |  |  | 68.4 | Directly / Minor | 83.0 | 16.5 |
| 6 | Day (2009) (29) | DJ |  | 70.0 |  |  |  |  |  |  |
| 7 | Farag (2015b) (30) | DJ; PHE; Olij |  | 70.0 |  |  | 68.4 | Partially / Minor | 75.0 | 3.4 |
| 8 | Frick (2010) (31) | DJ; PHE; Olij |  | 60.0 |  |  | 68.4 | Partially / Minor | 68.0 | 4.7 |
| 9 | Hektoen (2009) (32) | DJ; PHE |  | 90.0 |  |  | 26.5 | Partially / Potentially serious |  | 45.0 |
| 10 | Johansson (2008) (33) | DJ |  | 90.0 |  |  |  |  |  |  |
| 11 | Lee (2013) (34) | Olij |  |  |  |  |  |  | 90.0 |  |
| 12 | Ling (2008) (35) | DJ |  | 30.0 |  |  |  |  |  |  |
| 13 | McLean (2015) (36) | PHE; Olij; Huter; Winser |  | 90.0 |  | 94.0 | 73.7 | Directly / Minor | 95.0 | 9.9 |
| 14 | Mori (2017) (37) | Olij |  |  |  |  |  |  | 93.0 |  |
| 15 | OMAS (2008) (38) | DJ |  | 80.0 |  |  |  |  |  |  |
| 16 | Pega (2016) (39) | PHE; Olij |  |  |  |  | 84.2 | Directly / Minor | 83.0 | 0.9 |
| 17 | Poole (2015) (40) | Olij |  |  |  |  |  |  | 75.0 |  |
| 18 | Sach (2007) (41)^7^ | Davis; Olij | 100 |  | 92.0 |  |  |  | 89.0 | 5.7 |
| 19 | Smith (1998) (42) | RCN; Davis; Olij | 70.0 |  | 47.0 |  |  |  | 76.0 | 15.3 |
| 20 | van der Velde (2008) (43) | PHE |  |  |  |  | 52.6 | Directly / Minor |  |  |
| 21 | Wu (2010) (44) | PHE |  |  |  |  | 52.6 | Directly / Minor |  |  |
|  |  | *Average:* | 80.0 | 70.9 | 66.0 | 94.0 | 59.6 |  | 82.7 | 12.5 |
| **Abbreviation:** CHEC: Consensus on Health Economic Criteria; DJ: Dubas-Jakobczyk; FoF: fear of falling; HAM: home assessment and modification; NICE: National Institute for Health and Care Excellence; OMAS: Ontario Medical Advisory Secretariat; PHE: Public Health England; PS: public sector; QHES: Quality of Health Economic Studies; RCN: Royal College of Nursing; RCT: randomized controlled trial.  **Shading:** Evaluations shaded in light grey received consistent quality ranking across multiple reviews that applied a quantitative quality checklist. Consistent ranking is defined as receiving quality scores that are all above or below the average score for each checklist by study design. Evaluations in dark grey received inconsistent ranking.  ^1^ References for systematic reviews: RCN (45); Davis (46); DJ (47); PHE (48); Olij (49); Huter (50); Winser (51)  ^2^ Contents of the checklists are given in Table D in Supplementary material.  ^3^ Davis review removed item 4 from the QHES checklist to produce a maximum score of 99. But it also calculated the percentage which is reported here.  ^4^ This column reports the number of ‘Yes’ in a 19-item checklist given to a study by the PHE review. Potential options are: ‘Yes’; ‘No’; ‘Partly’; ‘Unclear’; ‘N/A’. PHE review added an extra item (item 8; see Table D in Supplementary material) to the original checklist.  ^5^ Categories for applicability: ‘Directly’; ‘Partially’; ‘Not’. Categories for general limitations: ‘Minor’; ‘Potentially serious’; ‘Very serious’.  ^6^ Olij review added an extra item (item 5; see Table D in Supplementary material) to the original checklist.  ^7^ This study was included as a decision model because it extrapolated the results of a trial over a lifetime horizon. | | | | | | | | | | |

# Reporting and methodological quality of systematic reviews

| **Table F** AMSTAR 2 checklist for reporting and methodological quality of systematic reviews (56). | | | | | | | |
| --- | --- | --- | --- | --- | --- | --- | --- |
| **Checklist item** | **Systematic review – publication year** | | | | | | |
|  | RCN (45) – 2005 | Davis (46) – 2010 | DJ (47) – 2017 | PHE (48) – 2018 | Olij (49) – 2018 | Huter (50) – 2018 | Winser (51) – 2019 |
| (1) Did the research questions and inclusion criteria include the components of PICO? (Response: Yes; No) | No (a) | Yes | Yes | Yes | Yes | Yes | Yes |
| (2) Did the report of the review contain an explicit statement that the review methods were established prior to the conduct of the review and did the report justify any significant deviations from the protocol? (Response: Yes; Partial Yes; No)^1^ | No (b) | Partial Yes (e) | Partial Yes (e) | Partial Yes (e) | Yes | Partial Yes (e) | Yes |
| (3) Did the review authors explain their selection of the study designs for inclusion in the review? (Response: Yes; No) | Yes | Yes | Yes | Yes | Yes | Yes | Yes |
| (4) Did the review authors use a comprehensive literature search strategy? (Response: Yes; Partial Yes; No)^2^ | Partial Yes (c) | Partial Yes (c) | Yes | Partial Yes (c) | Yes | Yes | Partial Yes (c) |
| (5) Did the review authors perform study selection in duplicate? (Response: Yes; No) | No | No | Yes | Yes | Yes | Yes | Yes |
| (6) Did the review authors perform data extraction in duplicate? (Response: Yes; No) | No | Yes | Yes | Yes | Yes | Yes | Yes |
| (7) Did the review authors provide a list of excluded studies and justify the exclusions? (Response: Yes; Partial Yes; No)^3^ | Partial Yes (d) | No | No | Yes | No | No | No |
| (8) Did the review authors describe the included studies in adequate detail? (Response states the number of data fields extracted; see manuscript Table 3).^4^ | No | Yes | Partial Yes | Yes | Partial Yes | Yes (j) | Yes |
| (9) Did the review authors use a satisfactory technique for assessing the risk of bias in individual studies that were included in the review? (Response: Yes; Partial Yes; No)^5^ | No (b) | Partial Yes | Yes | Yes | Partial Yes | Yes | Partial Yes |
| (10) Did the review authors report on the sources of funding for the studies included in the review? (Response: Yes; No) | No | Yes (f) | No | Yes (f) | Yes (f) | No | Yes (f) |
| (11) If meta-analysis was performed did the review authors use appropriate methods for statistical combination of results? (Response: Yes; No; No meta-analysis conducted) | No meta-an | No meta-an | No meta-an | No meta-an | No meta-an | No meta-an | No meta-an |
| (12) If meta-analysis was performed, did the review authors assess the potential impact of risk of bias in individual studies on the results of the meta-analysis or other evidence synthesis? (Response: Yes; No; No meta-analysis conducted) | No meta-an | No meta-an | No meta-an | No meta-an | No meta-an | No meta-an | No meta-an |
| (13) Did the review authors account for risk of bias in individual studies when interpreting/discussing the results of the review? (Response: Yes; No)^6^ | No | No | Yes (g) | No (h) | No (i) | Yes (k) | Yes |
| (14) Did the review authors provide a satisfactory explanation for, and discussion of, any heterogeneity observed in the results of the review? (Response: Yes; No) | No | Yes | Yes | Yes | Yes | Yes | Yes |
| (15) If they performed quantitative synthesis did the review authors carry out an adequate investigation of publication bias (small study bias) and discuss its likely impact on the results of the review? (Response: Yes; No; No meta-analysis conducted) | No meta-an | No meta-an | No meta-an | No meta-an | No meta-an | No meta-an | No meta-an |
| (16) Did the review authors report any potential sources of conflict of interest, including any funding they received for conducting the review? (Response: Yes; No) | Yes | Yes | Yes | Yes | Yes | Yes | Yes |
| **Number of ‘Yes’ or ‘Partial Yes’** | 4 | 10 | 11 | 12 | 11 | 11 | 12 |
| **Number of ‘Yes’** | 2 | 7 | 9 | 10 | 9 | 10 | 10 |
| **Abbreviation:** AMSTAR: assessment of multiple systematic reviews; Meta-an: meta-analysis; QHES: Quality of Health Economics Studies.  ^1^ For Partial Yes, included: review question; search strategy; inclusion/exclusion criteria; risk of bias assessment. For Yes, as for Partial Yes, plus protocol registration and specified: meta-analysis/synthesis plan; plan for investigating causes of heterogeneity; justification for any deviations from the protocol. See footnote 5 concerning the relevance of risk of bias to reviews of economic evaluations.  ^2^ For Partial Yes: searched at least two databases; provided keywords and/or search strategy; justified publication restrictions (e.g., language). For Yes, as for Partial Yes, plus done all of the following: searched the reference lists of included studies; searched trial/study registries (not relevant to this study); included/consulted content experts in the field; searched for grey literature where relevant; conducted search within 24 months of completion of the review.  ^3^ For Partial Yes, provided a list of all potentially relevant studies that were read in full-text form but excluded from the review. For Yes, justified the exclusion from the review of each potentially relevant study.  ^4^ The AMSTAR 2’s lists of details to be described by reviews did not adequately cover features relevant to economic evaluations. Hence, this item was judged based on the number of data fields extracted from evaluations as reported in the manuscript Table 3: Yes if 30 or more fields extracted; Partial Yes if 20-29 fields extracted; No if <20 fields extracted. An exception is the Huter review which deliberately extracted a limited number of data fields. This is given a Yes.  ^5^ In AMSTAR 2, this item chiefly concerns the risk of bias in intervention effectiveness estimate in randomised and non-randomised studies. Although clinical effectiveness is an important parameter for economic evaluation, a broader set of factors (e.g., costing of resource use) determine the credibility of economic outcomes. Therefore, Partial Yes was given if the review applied a methodological/reporting quality checklist to included evaluations; Yes was given if in addition to checklist, a narrative synthesis of methodological features was conducted.  ^6^ See footnote 5 concerning the relevance of risk of bias to reviews of economic evaluations.  (a) Does not specify the intervention and comparator.  (b) Does not state the method of risk of bias (i.e., methodological quality) assessment. The review states that the Drummond checklist was applied but the scores are not reported.  (c) Does not mention searching grey literature and/or included studies’ reference lists.  (d) Results suggest that 14 full texts were assessed, and Table 18 presents 8 articles; but the section ‘Excluded studies’ only discusses 4 excluded articles. Hence, the review gives the exclusion reason only for a subset of excluded studies.  (e) Does not mention registering the review protocol.  (f) Funding source or conflict of interest was an item in the checklist used by the review to assess the reporting/methodological quality of included evaluations.  (g) Evaluation outcomes were grouped by categories of methodological quality of evaluations: ‘Good’, ‘Moderate’ and ‘Poor’ based on Drummond checklist score (see Table D above).  (h) Applied checklist and listed several methodological caveats per evaluation but these were not mentioned when describing the ‘key messages’ by intervention type in Section 5.3.  (i) Does not discuss the methodological quality checklist scores when describing the evaluation outcomes.  (j) Given a Yes because the review deliberately limited the number of data fields extracted; eight fields were extracted as the result.  (k) Methodological features of individual evaluations described in Table S1. | | | | | | | |

# References

1. Campbell AJ, Robertson MC, La Grow SJ, Kerse NM, Sanderson GF, Jacobs RJ, et al. Randomised controlled trial of prevention of falls in people aged > or =75 with severe visual impairment: the VIP trial. BMJ. 2005;331(7520):817.

2. Cockayne S, Rodgers S, Green L, Fairhurst C, Adamson J, Scantlebury A, et al. Clinical effectiveness and cost-effectiveness of a multifaceted podiatry intervention for falls prevention in older people: a multicentre cohort randomised controlled trial (the REducing Falls with ORthoses and a Multifaceted podiatry intervention trial). Health technology assessment (Winchester, England). 2017;21(24):1-198.

3. Davis JC, Marra CA, Robertson MC, Khan KM, Najafzadeh M, Ashe MC, et al. Economic evaluation of dose-response resistance training in older women: a cost-effectiveness and cost-utility analysis. Osteoporos International. 2011;22(5):1355-66.

4. Davis JC, Marra CA, Robertson MC, Najafzadeh M, Liu‐Ambrose T. Sustained economic benefits of resistance training in community‐dwelling senior women. Journal of the American Geriatrics Society. 2011;59(7):1232-7.

5. Farag I, Howard K, Hayes AJ, Ferreira ML, Lord SR, Close JT, et al. Cost-effectiveness of a Home-Exercise Program Among Older People After Hospitalization. Journal of the American Medical Directors Association. 2015;16(6):490-6.

6. Farag I, Sherrington C, Hayes A, Canning CG, Lord SR, Close JCT, et al. Economic evaluation of a falls prevention exercise program among people With Parkinson's disease. Movement disorders. 2016;31(1):53-61.

7. Fletcher E, Goodwin VA, Richards SH, Campbell JL, Taylor RS. An exercise intervention to prevent falls in Parkinson's: an economic evaluation. BMC Health Serv Res. 2012;12:426.

8. Hendriks MR, Evers SM, Bleijlevens MH, van Haastregt JC, Crebolder HF, van Eijk JTM. Cost-effectiveness of a multidisciplinary fall prevention program in community-dwelling elderly people: a randomized controlled trial (ISRCTN 64716113). International journal of technology assessment in health care. 2008;24(2):193-202.

9. Irvine L, Conroy SP, Sach T, Gladman JRF, Harwood RH, Kendrick D, et al. Cost-effectiveness of a day hospital falls prevention programme for screened community-dwelling older people at high risk of falls. Age and Ageing. 2010;39(6):710-6.

10. Isaranuwatchai W, Perdrizet J, Markle-Reid M, Hoch JS. Cost-effectiveness analysis of a multifactorial fall prevention intervention in older home care clients at risk for falling. BMC geriatrics. 2017;17(1):199.

11. Jenkyn KB, Hoch JS, Speechley M. How much are we willing to pay to prevent a fall? Cost-effectiveness of a multifactorial falls prevention program for community-dwelling older adults. Canadian Journal on Aging/La Revue canadienne du vieillissement. 2012;31(2):121-37.

12. Kenkre JE, Allan TF, Tobias RS, Parry DJ, Bryan S, Carter YH. Breaking bones, breaking budgets: a clinical and economic evaluation of a prospective, randomized, practice controlled, intervention study in the prevention of accidents in primary care. Family practice. 2002;19(6):675-81.

13. Li F, Harmer P. Economic Evaluation of a Tai Ji Quan Intervention to Reduce Falls in People With Parkinson Disease, Oregon, 2008-2011. Prev Chronic Dis. 2015;12:E120.

14. Patil R, Kolu P, Raitanen J, Valvanne J, Kannus P, Karinkanta S, et al. Cost-effectiveness of vitamin D supplementation and exercise in preventing injurious falls among older home-dwelling women: findings from an RCT. Osteoporosis International. 2016;27(1):193-201.

15. Peeters GMEE, Heymans MW, de Vries OJ, Bouter LM, Lips P, van Tulder MW. Multifactorial evaluation and treatment of persons with a high risk of recurrent falling was not cost-effective. Osteoporosis International. 2011;22(7):2187-96.

16. Polinder S, Boye ND, Mattace-Raso FU, Van der Velde N, Hartholt KA, De Vries OJ, et al. Cost-utility of medication withdrawal in older fallers: results from the improving medication prescribing to reduce risk of FALLs (IMPROveFALL) trial. BMC Geriatr. 2016;16(1):179.

17. Rizzo JA, Baker DI, McAvay G, Tinetti ME. The cost-effectiveness of a multifactorial targeted prevention program for falls among community elderly persons. Medical care. 1996:954-69.

18. Robertson MC, Devlin N, Gardner MM, Campbell AJ. Effectiveness and economic evaluation of a nurse delivered home exercise programme to prevent falls. 1: Randomised controlled trial. BMJ. 2001;322(7288):697-701.

19. Robertson MC, Gardner MM, Devlin N, McGee R, Campbell AJ. Effectiveness and economic evaluation of a nurse delivered home exercise programme to prevent falls. 2: Controlled trial in multiple centres. BMJ. 2001;322(7288):701-4.

20. Robertson MC, Devlin N, Scuffham P, Gardner MM, Buchner DM, Campbell AJ. Economic evaluation of a community based exercise programme to prevent falls. J Epidemiol Community Health. 2001;55(8):600-6.

21. Sach TH, Logan PA, Coupland CA, Gladman JR, Sahota O, Stoner-Hobbs V, et al. Community falls prevention for people who call an emergency ambulance after a fall: an economic evaluation alongside a randomised controlled trial. Age Ageing. 2012;41(5):635-41.

22. Salkeld G, Cumming RG, O'Neill E, Thomas M, Szonyi G, Westbury C. The cost effectiveness of a home hazard reduction program to reduce falls among older persons. Australian and New Zealand journal of public health. 2000;24(3):265-71.

23. Timonen L, Rantanen T, Mäkinen E, Timonen T, Törmäkangas T, Sulkava R. Cost analysis of an exercise program for older women with respect to social welfare and healthcare costs: a pilot study. Scandinavian journal of medicine & science in sports. 2008;18(6):783-9.

24. Albert SM, Raviotta J, Lin CJ, Edelstein O, Smith KJ. Cost-effectiveness of a statewide falls prevention program in Pennsylvania: Healthy Steps for Older Adults. The American journal of managed care. 2016;22(10):638-44.

25. Beard J, Rowell D, Scott D, van Beurden E, Barnett L, Hughes K, et al. Economic analysis of a community-based falls prevention program. Public Health. 2006;120(8):742-51.

26. Carande-Kulis V, Stevens JA, Florence CS, Beattie BL, Arias I. A cost-benefit analysis of three older adult fall prevention interventions. J Safety Res. 2015;52:65-70.

27. Church J, Goodall S, Norman R, Haas M. An economic evaluation of community and residential aged care falls prevention strategies in NSW. New South Wales public health bulletin. 2011;22(3-4):60-8.

28. Church J, Goodall S, Norman R, Haas M. The cost-effectiveness of falls prevention interventions for older community-dwelling Australians. Aust N Z J Public Health. 2012;36(3):241-8.

29. Day L, Hoareau E, Finch C, Harrison JE, Segal L, Bolton TG, et al. Modelling the impact, cost and benefits of falls prevention measures to support policy-makers and program planners. 2009.

30. Farag I, Howard K, Ferreira ML, Sherrington C. Economic modelling of a public health programme for fall prevention. Age Ageing. 2015;44(3):409-14.

31. Frick KD, Kung JY, Parrish JM, Narrett MJ. Evaluating the cost-effectiveness of fall prevention programs that reduce fall-related hip fractures in older adults. J Am Geriatr Soc. 2010;58(1):136-41.

32. Hektoen LF, Aas E, Luras H. Cost-effectiveness in fall prevention for older women. Scand J Public Health. 2009;37(6):584-9.

33. Johansson P, Sadigh S, Tillgren P, Rehnberg C. Non-pharmaceutical prevention of hip fractures - a cost-effectiveness analysis of a community-based elderly safety promotion program in Sweden. Cost effectiveness and resource allocation : C/E. 2008;6:11.

34. Lee RH, Weber T, Colon-Emeric C. Comparison of cost-effectiveness of vitamin D screening with that of universal supplementation in preventing falls in community-dwelling older adults. Journal of the American Geriatrics Society. 2013;61(5):707-14.

35. Ling C, Henderson S, Henderson R, Henderson M, Pedro T, Pang L. Cost benefit considerations of preventing elderly falls through environmental modifications to homes in Hana, Maui. Hawaii medical journal. 2008;67(3):65.

36. McLean K, Day L, Dalton A. Economic evaluation of a group-based exercise program for falls prevention among the older community-dwelling population. BMC Geriatr. 2015;15:33.

37. Mori T, Crandall C, Ganz DA. Cost-effectiveness of combined oral bisphosphonate therapy and falls prevention exercise for fracture prevention in the USA. Osteoporosis international. 2017;28(2):585-95.

38. Ontario Medical Advisory Secretariat. The Falls/fractures Economic Model in Ontario Residents aged 65 years and over (FEMOR). Ontario health technology assessment series. 2008;8(6):1.

39. Pega F, Kvizhinadze G, Blakely T, Atkinson J, Wilson N. Home safety assessment and modification to reduce injurious falls in community-dwelling older adults: cost-utility and equity analysis. Injury prevention : journal of the International Society for Child and Adolescent Injury Prevention. 2016;22(6):420-6.

40. Poole CD, Smith J, Davies JS. Cost-effectiveness and budget impact of Empirical vitamin D therapy on unintentional falls in older adults in the UK. BMJ open. 2015;5(9):e007910.

41. Sach TH, Foss AJ, Gregson RM, Zaman A, Osborn F, Masud T, et al. Falls and health status in elderly women following first eye cataract surgery: an economic evaluation conducted alongside a randomised controlled trial. Br J Ophthalmol. 2007;91(12):1675-9.

42. Smith RD, Widiatmoko D. The cost-effectiveness of home assessment and modification to reduce falls in the elderly. Australian and New Zealand journal of public health. 1998;22(4):436-40.

43. van der Velde N, Meerding WJ, Looman CW, Pols HA, van der Cammen TJ. Cost effectiveness of withdrawal of fall-risk-increasing drugs in geriatric outpatients. Drugs & aging. 2008;25(6):521-9.

44. Wu S, Keeler EB, Rubenstein LZ, Maglione MA, Shekelle PG. A cost-effectiveness analysis of a proposed national falls prevention program. Clinics in geriatric medicine. 2010;26(4):751-66.

45. Royal College of Nursing. Clinical practice guideline for the assessment and prevention of falls in older people. Clinical Practice Guidelines. 2005;London: Royal College of Nursing.

46. Davis JC, Robertson MC, Ashe MC, Liu-Ambrose T, Khan KM, Marra CA. Does a home-based strength and balance programme in people aged > or =80 years provide the best value for money to prevent falls? A systematic review of economic evaluations of falls prevention interventions. Br J Sports Med. 2010;44(2):80-9.

47. Dubas-Jakóbczyk K, Kocot E, Kissimova-Skarbek K, Huter K, Rothgang H. Economic evaluation of health promotion and primary prevention actions for older people—a systematic review. The European Journal of Public Health. 2017;27(4):670-9.

48. Public Health England. A structured literature review to identify cost-effective interventions to prevent falls in older people living in the community. Public Health England. 2018.

49. Olij BF, Ophuis RH, Polinder S, Van Beeck EF, Burdorf A, Panneman MJ, et al. Economic evaluations of falls prevention programs for older adults: a systematic review. Journal of the American Geriatrics Society. 2018;66(11):2197-204.

50. Huter K, Dubas-Jakóbczyk K, Kocot E, Kissimova-Skarbek K, Rothgang H. Economic evaluation of health promotion interventions for older people: do applied economic studies meet the methodological challenges? Cost Effectiveness and Resource Allocation. 2018;16(1):14.

51. Winser SJ, Chan HTF, Ho L, Chung LS, Ching LT, Felix TKL, et al. Dosage for cost-effective exercise-based falls prevention programs for older people: a systematic review of economic evaluations. Annals of physical and rehabilitation medicine. 2020;63(1):69-80.

52. Drummond MF, Sculpher MJ, Claxton K, Stoddart GL, Torrance GW. Methods for the economic evaluation of health care programmes: Oxford university press; 2015.

53. Ofman JJ, Sullivan SD, Neumann PJ, Chiou C-F, Henning JM, Wade SW, et al. Examining the value and quality of health economic analyses: implications of utilizing the QHES. Journal of Managed Care Pharmacy. 2003;9(1):53-61.

54. National Institute for Health and Care Excellence. Developing NICE guidelines: the manual Appendix H. London: NICE. 2014.

55. Evers S, Goossens M, de Vet H, van Tulder M, Ament A. Criteria list for assessment of methodological quality of economic evaluations: Consensus on Health Economic Criteria. Int J Technol Assess Health Care. 2005;21(2):240-5.

56. Shea BJ, Reeves BC, Wells G, Thuku M, Hamel C, Moran J, et al. AMSTAR 2: a critical appraisal tool for systematic reviews that include randomised or non-randomised studies of healthcare interventions, or both. bmj. 2017;358.
